# Live *Akkermansia muciniphila* boosts dendritic cell retinoic acid synthesis to modulate IL-22 activity and mitigate colitis in mice

**DOI:** 10.1186/s40168-024-01995-7

**Published:** 2024-12-30

**Authors:** Hongbin Liu, Ruo Huang, Binhai Shen, Chongyang Huang, Qian zhou, Jiahui Xu, Shengbo Chen, Xinlong Lin, Jun Wang, Xinmei Zhao, Yandong Guo, Xiuyun Ai, Yangyang Liu, Ye Wang, Wendi Zhang, Fachao Zhi

**Affiliations:** 1 Department of Gastroenterology, Guangdong Provincial Key Laboratory of Gastroenterology, Institute of Gastroenterology of Guangdong Province, Nanfang Hospital, Southern Medical University, Guangzhou, China; 2 Department of Gastroenterology, The Second Affiliated Hospital of Guangzhou University of Chinese Medicine, Guangzhou, China; 3 Department of Gastroenterology, The Second Affiliated Hospital of Guangzhou Medical University, Guangzhou, China; 4 Department of Gastroenterology, Institute of Digestive Diseases, The Affiliated Qingyuan Hospital (Qingyuan People’s Hospital), Guangzhou Medical University, Qingyuan, China; 5 Huiqiao Medical Center, Nanfang Hospital, Southern Medical University, Guangzhou, China; 6 Guangzhou ZhiYi Biotechnology Co., Ltd, Guangzhou, China

**Keywords:** *Akkermansia muciniphila*, Ulcerative colitis, Retinoic acid, Dendritic cells, IL-22, Group 3 innate lymphoid cells, STAT3

## Abstract

**Background:**

The interplay between gut microbiota and immune responses is crucial in ulcerative colitis (UC). Though *Akkermansia muciniphila* (Akk) shows therapeutic potential, the mechanisms remain unclear. This study sought to investigate differences in therapeutic efficacy among different forms or strains of Akk and elucidate the underlying mechanisms.

**Results:**

Employing a dextran sulfate sodium (DSS)-induced colitis mouse model, we assessed Akk’s impact on colitis using cellular cytokine analysis, immune phenotyping, proteomics, and biochemical methods. Our results suggest that treatment with live Akk effectively reduced colitis in the DSS-induced model, whereas heat-inactivated Akk did not yield the same results. Notably, Akk exhibited protective properties by promoting the secretion of IL-22 by Group 3 innate lymphoid cells (ILC3s), as evidenced by the absence of protection in IL-22 knockout mice. Additionally, Akk augmented the population of CD103^+^CD11b^−^ dendritic cells (DCs) and enhanced their retinoic acid (RA) synthesis through the modulation of RALDH2, a crucial enzyme in RA metabolism. The depletion of RALDH2 in DCs diminished Akk’s protective properties and impaired IL-22-mediated mucosal healing. Mechanistically, Akk activated RA production in DCs by enhancing the JAK2-STAT3 signaling pathway. Additionally, various strains of Akk may exhibit differing abilities to alleviate colitis, with the novel strain Am06 derived from breast milk showing consistent efficacy similar to the reference strain.

**Conclusions:**

In summary, our findings indicate that certain strains of Akk may mitigate colitis through the promotion of RA synthesis and IL-22 secretion, underscoring the potential efficacy of Akk as a therapeutic intervention for the management of UC.

Video Abstract

**Supplementary Information:**

The online version contains supplementary material available at 10.1186/s40168-024-01995-7.

## Background

Ulcerative colitis (UC) is a chronic inflammatory disease primarily targeting the colon [1]. While its exact cause is unknown, a disruption in gut microbiota precedes disease onset, implicating microbe involvement in UC [2]. Despite uncertainties about the link between gut microbiota and UC, there is a growing interest in microbiota-based interventions [3]. Previous studies have mainly focused on pathogens associated with UC [4], while specific potential beneficial bacteria have been found to be involved in regulating host immunity and mitigating colonic inflammation [5, 6]. However, the mechanisms behind bacterial immune regulation in the gut remain largely undetermined.

Retinoic acid (RA), vitamin A’s bioactive metabolite, is crucial for various physiological processes [7]. It assists in the differentiation of regulatory T cells (Tregs) crucial for intestinal homeostasis, a process facilitated by CD103^+^ Dendritic Cells (DCs). These DCs efficiently synthesize RA by expressing the critical enzyme, retinaldehyde dehydrogenase 2 (RALDH2) [8, 9]. Moreover, RA also plays a crucial role in regulating innate immune cells. Specifically, RA aids in the conversion of innate lymphoid cells from type 1 (ILC1s) to type 3 (ILC3s) and promotes the expression of IL-22, a vital cytokine involved in mucosal repair [10–12]. In line with this, by enhancing the secretion of IL-22, RA effectively mitigates colitis induced by dextran sulfate sodium (DSS) [12]. A noticeable decrease in RALDH activity in CD103^+^ DCs has been observed in mice with colitis and individuals with UC [13, 14]. Consequently, the restoration of CD103^+^ DC subsets and their RALDH activity could potentially form a therapeutic strategy for UC [13, 15]. Despite reports of microbiota’s role in RA metabolism [16, 17], an understanding of how specific symbiotic strains regulate RA synthesis in DCs remains scant.

*Akkermansia muciniphila* (Akk), a commensal bacterium commonly found in healthy individuals, is significantly decreased in abundance in multiple UC studies [18, 19]. Akk has shown probiotic potential for immune and metabolic disorders [20]. Notably, heat-inactivated Akk has demonstrated enhanced efficacy in multiple studies involving mice and humans [21, 22]. Despite its viability being highlighted as crucial in recent research [23, 24], studies on Akk’s therapeutic role in colitis yield contrasting results, potentially due to strain variations [25, 26]. Therefore, detailed research on the mode of action, strain specificity, and cell viability necessity is requisite.

This study unveils a novel mechanism that elucidates the anti-inflammatory effects of Akk on colitis in a preclinical model. The supplementation of live Akk, as opposed to its heat-inactivated forms, demonstrates the potential to alleviate colitis. Further analyses performed in vivo and in vitro demonstrate that Akk stimulates IL-22 secretion from ILC3s by replenishing the population and RA synthesis activity of diminished CD103^+^ DCs in colitis mice, ultimately promoting mucosal healing. These findings enhance our comprehension of Akk’s pathophysiological roles, offering a robust theoretical foundation for the potential utilization of probiotics in the treatment of UC.

## Methods

### Culture of Akk

The Akk reference strain (ATCC BAA-835) was acquired from the American Type Culture Collection (ATCC; Manassas, USA), while Am03 and Am06 were donated by Zhiyi Biotech (Guangzhou, China). All three species were cultivated using a Brain Heart Infusion (BHI) medium containing 0.4% N-Acetylglucosamine and 0.5% L-Threonine under anaerobic conditions at 37 °C for 24 h. After centrifugation, the Akk pellets were resuspended in sterile phosphate-buffered saline (PBS). These pellets were then subjected to heat treatment at either 70 °C for 30 min or 100 °C for 15 min, to produce pasteurized Akk or heat-killed Akk. To detect the synthesis of RA, retinaldehyde (ROL) was added to the Akk culture system after 24 h of culture, achieving a final concentration of 1 μM. The culture was incubated for another four hours under light avoidance and anaerobic conditions, then centrifuged at 4000 × *g* for 10 min. Then, the bacterial supernatant was collected for subsequent analysis.

### Mouse model

Four to 5-week-old Male C57BL/6 J wild-type (WT) mice were procured from the Vital River Laboratory Animal Co. (Zhejiang, China). Dr. Qian Zhou kindly donated IL-22^-/-^ mice. Upon request, *Raldh2*^*fl/fl*^ mice were developed at Cyagen Biosciences (Jiangsu, China). We acquired CD11c cre mice from the Shanghai Model Organisms Center (Shanghai, China). By crossing female *Raldh2*^*fl/fl*^ mice with male CD11c cre transgenic mice, we produced *Raldh2*^*ΔDC*^ mice, which express the Cre gene. *Raldh2*^*fl/fl*^ mice were used as controls. All the mice were housed under specific pathogen-free (SPF) conditions at the animal facility of Nanfang Hospital. The Southern Medical University’s Ethics Committee for Animal Experiments granted ethical approval for our study (Approval Nos: K2019090) in line with institutional animal welfare guidelines. Before setting up the model, the mice underwent a 14-day bacterial gavage pre-treatment, receiving either live Akk, pasteurized Akk, heat-killed Akk , or 200 µL PBS. Dextran sodium sulfate (DSS; MP Biomedical, USA) was used to induce both chronic and acute colitis. The chronic model comprised three cycles of a 7-day 2% DSS, followed by a 14-day sterilized tap water, alongside the bacterial gavage or not. The acute model exposed the mice to a 2.5% DSS for 7 days with/without the bacterial gavage. We adopted a modified protocol for the daily oral administration of 100 μg of RA per mouse [17]. We monitored disease progression daily by recording body weight and the disease activity index (DAI), calculated from weight loss, fecal consistency, and rectal bleeding scores.

### Isolation of colonic lamina propria lymphocytes (LPLs)

The mouse colon tissue was prepared under aseptic conditions with the removal of the Peyer’s patches. The tissue was then opened longitudinally and cleansed with PBS. Following that, the tissue was segmented into 0.5 cm fragments and situated in a D-Hanks buffer that contained 5% fetal bovine serum (FBS), 20 mM HEPES, 5 mM Ethylene Diamine Tetraacetic Acid Disodium (EDTA-Na_2_), and 2 mM dithiothreitol (DTT). These fragments were incubated twice at 37 °C for twenty-minute intervals, agitating at 220 rpm. Following this, the colon fragments were submitted to a 30-min incubation on a shaker at 37 °C in Hanks buffer with 5% FBS, 500 µg/mL collagenase IV (Vetec, USA), 50 µg/mL DNase I (Roche, UK), and 0.5 U/mL Dispase II (Yeasen, China). The mixture was passed through a 70-µm cell strainer before being centrifuged at 300 × *g* for 5 min. Immune cells from the colonic lamina propria (CLP) were then purified with a density gradient centrifugation technique using a 40% and 80% gradient Percoll-IMDM solution.

### Flow cytometry

After rinsing in the Staining buffer (2% FBS in PBS), cells were exposed to the Fc receptor-blocking antibody (BioLegend, USA). Single-cell suspensions were then stained with specific antibodies to surface markers at 4 °C for 30 min. For the Alderfluor assay, the single-cell suspension was prepared following the manufacturer’s protocols, using 5 μL of BAAA with or without Diethylaminobenzaldehyde (DEAB) for 30 min at 37 °C. In the case of cytokine staining, the single-cell suspension was first treated with a protein transport inhibitor cocktail for five hours at 37 °C, then followed by the full staining process. Post-surface protein staining, cells were fixed using a fixation and permeabilization staining buffer set (Invitrogen, USA) at 4 °C in light-protected conditions for around 20 min. The cell suspensions were later stained with antibodies against intracellular antigens and cytokines, labeled with various fluorescent dyes, and incubated in light-protected conditions at 4 °C for 30 min. Flow cytometry analysis and sorting were done using the FACS Aria III (BD, USA).

### Statistical analysis

The data from this study was derived from three independent experiments. Unless otherwise noted, results are expressed as mean ± standard error of the mean (SEM). Statistical analyses were conducted using GraphPad Prism version 9.0. For comparisons involving independent samples, an independent samples *t*-test was applied when the variance homogeneity assumption was satisfied; if not, the Satterthwaite approximate *t*-test was utilized. For analyses involving multiple independent samples, a one-way analysis of variance (ANOVA) was conducted, followed by post hoc multiple comparison tests using the Tukey method. If the variance homogeneity assumption was not met, the Dunnett T3 method was employed. Survival data were analyzed with the log-rank (Mantel-Cox) test, using a Bonferroni-adjusted significance threshold of 0.005. For repeated measures data, a repeated measures ANOVA was used. In the figures, * indicates *P* < 0.05, **P < 0.01, ***P < 0.001, and ****P < 0.0001, denoting statistically significant differences.

## Results

### Live Akk alleviates DSS‐induced colitis

**Fig. 1:**
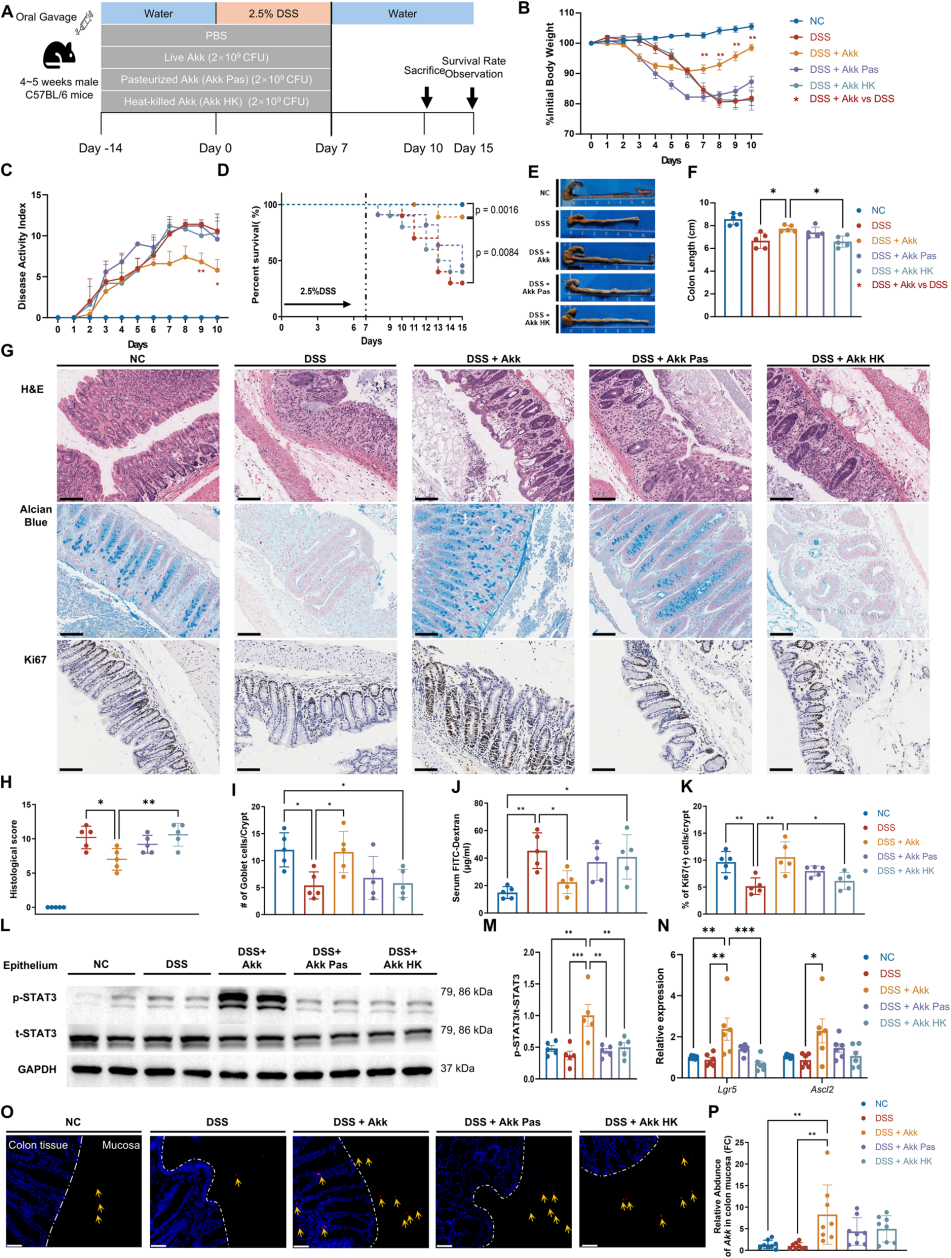
Live Akk alleviates DSS-induced acute colitis. **A** Mice were assigned to receive either drinking water for 14 days followed by 2.5% DSS for 7 days and then drinking water again for 3 or 8 days for survival studies, or solely plain water for 24 or 29 days for survival analysis (*n* = 5; 10 for survival). Prior to and during DSS treatment, groups were orally administered live, pasteurized, or heat-killed Akk, or PBS. **B–D** Percentage of weight loss (**B**), disease activity index (DAI) (**C**), and survival status (**D**) were assessed daily for each treatment group. **E**, **F** Representative images of the colons (**E**) and the assessment of colon length (**F**) from different treatment groups. **G**–**I** Representative images of the H&E-stained(upper), the Alcian Blue-stained colon sections (middle), and IHC images of Ki67 immunostaining (below) in the colon tissues of different treatment groups (scale bars 100 μm). Histological score (**H**) and goblet cells in the crypts (**I**) were assessed in the indicated groups. **J** Intestinal permeability of the relevant groups was determined by FITC-dextran level in serum. **K** Quantitative analysis of the percentage of Ki67^+^ cells in the crypts. **L**, **M** Representative immunoblot images of p-STAT3 and t-STAT3 in colon epithelial protein from different treatment groups (**L**). The protein levels of phosphorylated-STAT3 were quantified relative to total-STAT3 (**M**). **N** Expression of epithelial stem cell markers (*Lgr5* and *Ascl2*) in the indicated groups (*n* = 8). **O**, **P** The relative abundance of Akk in the colonic mucosa was determined by q-PCR (O) (*n* = 8). Confocal microscopy of Akk (P): Bacteria (red) are highlighted by a yellow arrow; DAPI staining appears in blue (scale bar 50 μm). *P < 0.05, **P < 0.01, ***P < 0.001

To determine the impact of Akk on colitis, mice were administered PBS, live Akk, pasteurized Akk, or heat-killed Akk, followed by DSS-induced acute colitis (Fig. 1A). Live Akk treatment led to less weight loss, improved disease activity index (DAI) (Fig. 1B, C), marginally enhanced survival (Fig. 1D, *P* = 0.00814) and substantially reduced DSS-induced intestinal damage, including severe colon shortening and an increased histological score (Fig. 1E–H). However, these benefits were absent in mice treated with heat-inactivated Akk.

DSS-induced colitis notably impairs the intestinal mucosal barrier, leading to goblet cells’ loss and increased intestinal permeability (Fig. 1G, I, J). However, these effects were mitigated with live Akk. The activation of signaling pathways like STAT3 is fundamental for the colonic mucosa’s regenerative capacity in countering injury-induced inflammation and disorders [27, 28]. Treatment with live Akk noticeably stimulates crypt proliferation (Fig. 1G, K), phosphorylated STAT3 (p-STAT3) expression (Fig. 1L, M), and increased epithelial stem cell markers *Lgr5* and *Ascl2* (Fig. 1N). Notably, these benefits diminish with heat inactivation of Akk.

Additionally, live Akk ameliorated weight loss and DAI in a chronic colitis model induced by three DSS cycles (Fig. S 1A–D). Only the live Akk group showed colon shortening improvement and better histopathological scoring (Fig. S 1E–H), indicating the necessity for living Akk to mitigate DSS-induced colitis. Consistently, lower doses of live Akk did not produce beneficial effects (Fig. S2A–F). However, administering live Akk culture’s supernatant didn't change results (Fig. S2G–L), potentially due to interference by the Akk culture system's animal constituents. Using qPCR assays, a significant increase in Akk abundance was observed in the live Akk group, indicating a correlation between Akk colonization and therapeutic efficacy (Fig. 1O). Further analysis using fluorescence in situ hybridization (FISH) confirmed the profound proximity of Akk to the colonic mucosa in the live Akk-treated group. Additionally, increased internalization of Akk by the cells within the colonic lamina propria (CLP) was observed in the group treated with live Akk (Fig. 1P). These findings affirm that live Akk, rather than other forms, effectively improves intestinal mucosal barrier and epithelial regeneration repair in mice with colitis.

### Akk reduced colonic inflammation and upregulated IL-22 expression by ILC3s

**Fig. 2:**
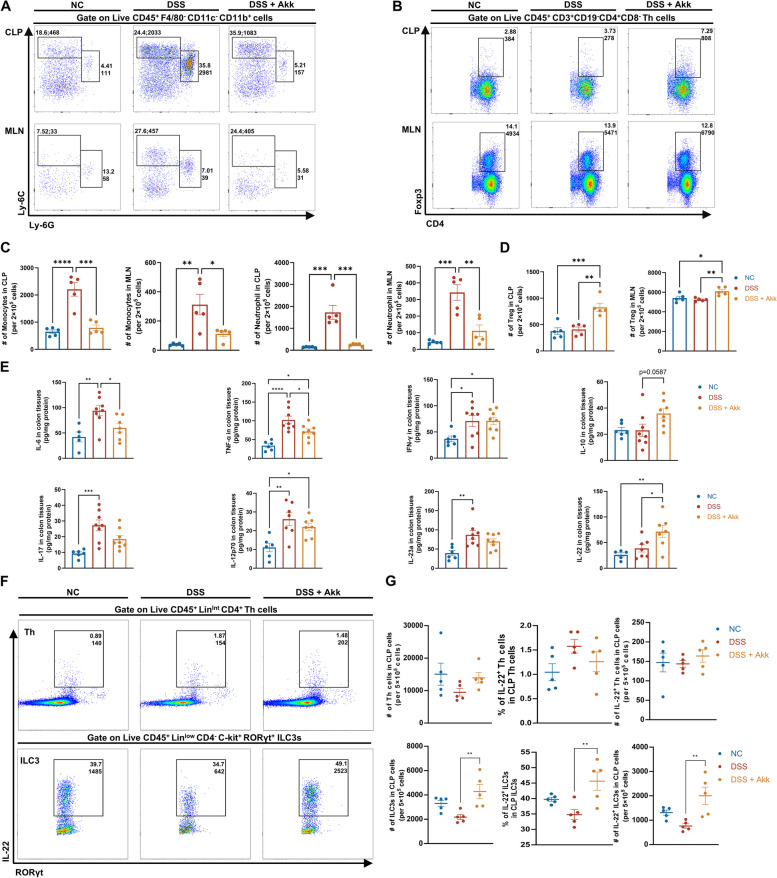
Akk reduces colonic inflammation and upregulates IL-22 expression by ILC3 cells. **A**–**D** Representative flow plots of CLP (top) and MLN (bottom) monocytes, neutrophils (**A**), and Tregs (**B**) in untreated mice and acute DSS-treated mice with gavage of PBS or Akk. Frequencies and absolute numbers of these specific populations **(C&D)** were determined (*n* = 5). **E** Cytokine levels in colonic proteins from relevant groups were assessed by ELISA (*n* = 6–8). **F, G** FACS analysis of total numbers of ILC3 cells **(F)**, the frequencies and absolute numbers of CLP IL-22^+^ ILC3s and IL-22^+^ Th cells from the indicated mice **(G)** (*n* = 5). *P < 0.05, **P < 0.01, ***P < 0.001, ****P < 0.0001

Considering the complex interplay between gut microbiota and the immune system, we investigated Akk’s impact on immune cells and cytokine profiles associated with inflammation. Through flow cytometry, we assessed immune cells in CLP and mesenteric lymph nodes (MLN) in acute colitis mice (Fig. S3A, B). Akk treatment significantly modified inflammatory monocytes and neutrophils (Fig. 2A, C), and notably increased Foxp3^+^ Tregs (Fig. 2B, D), without affecting CTL and B cells (Fig. S3C). ELISA on colon tissue homogenates showed Akk significantly reduced pro-inflammatory cytokines TNF-α and IL-6, while slightly enhancing anti-inflammatory IL-10 production (*P* = 0.0587) without changing IL-17 levels (Fig. 2E). Moreover, Akk treatment elevated IL-22 levels (Fig. 2E), a key cytokine for tissue repair and barrier function [29], despite not significantly augmenting levels of common drivers of IL-22 expression, IL-12, or IL-23 [30]. This was accompanied by an increase in IL-22-producing colonic ILC3s, but not Th cells (Fig. 2F, G and Fig. S3D). These results suggest Akk attenuates colonic inflammation by reducing immune cell infiltration and modulating cytokine profiles, with IL-22 from ILC3s playing a role in Akk’s protective effects against colitis in mice.

### The protective effect of Akk is mediated by the secretion of IL-22 by ILC3

**Fig. 3:**
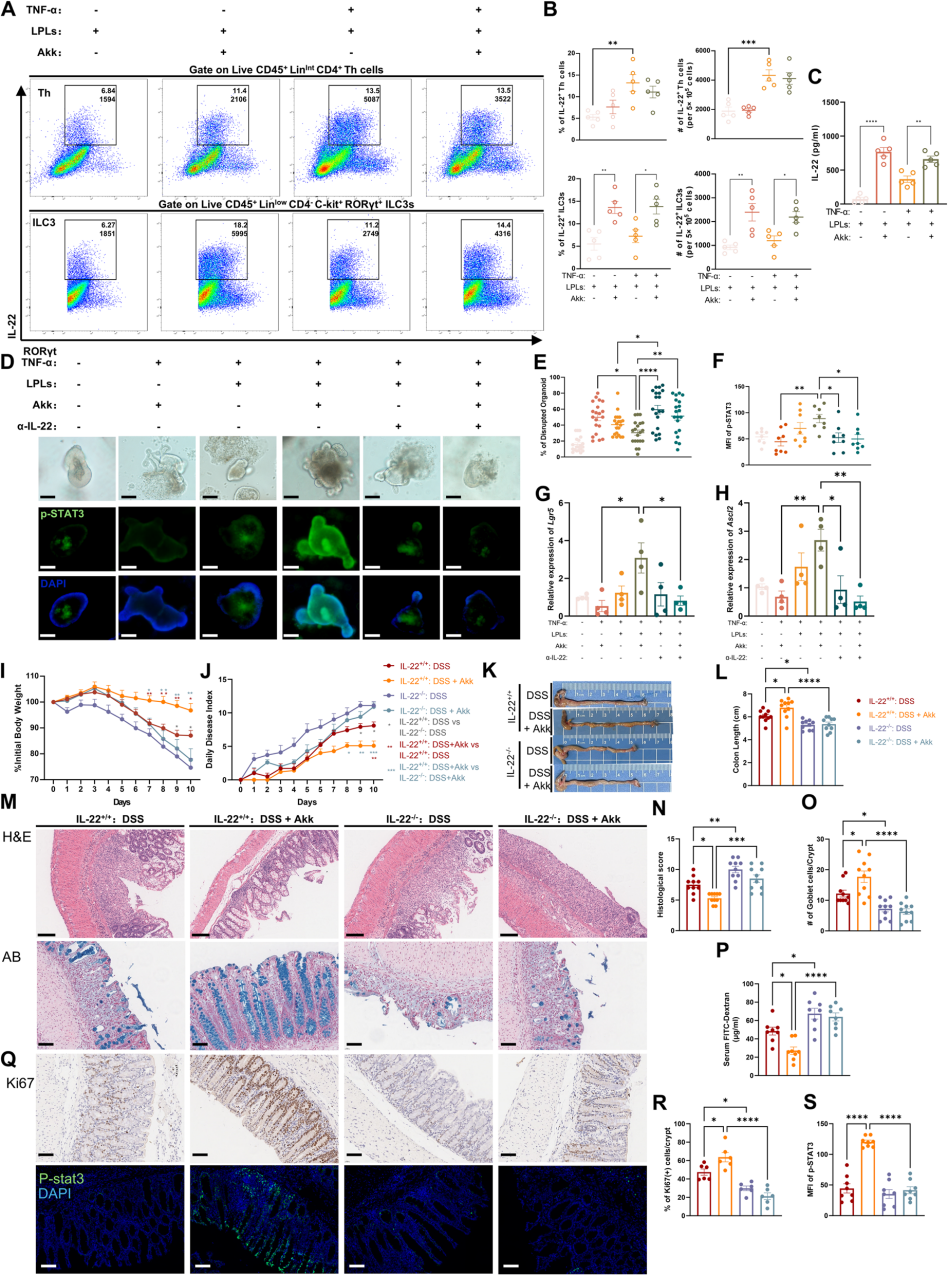
The protective effect of Akk is mediated by the secretion of IL-22 by ILC3 cells. **A**–**C** Colonic LPLs were treated with or without TNF-α or Akk (*n* = 5) for 12 h. The representative image of flow cytometric analysis for IL-22^+^ ILC3 cells and IL-22^+^ Th cells (**A**), including the frequencies and absolute numbers of these specific populations among different treatment groups (**B**). The IL-22 expression of cultured supernatants in these four groups was determined by ELISA (**C**). **D**–**H** Organoids were cultured with or without Akk or LPLs in the presence or absence of TNF-α or anti-IL-22 for 12 h. The morphologies of organoids (upper) and the immunofluorescence analysis of p-STAT3 (below; p-STAT3 staining in green, DAPI staining in blue; scale bars 100 μm) were evaluated by light or fluorescence microscopy (**D**). The relative number of organoids with altered morphology (**E**) (*n* = 4, randomly select five fields of view for observation in each well) and the average fluorescence intensity of p-STAT3 (**F**) were quantitatively assessed (*n* = 8 organoids per group). The mRNA levels of *Lgr5* (**G**) and *Ascl2* (**H**) in colonic organoids from different treatment groups were determined (*n* = 4). **I**–**J** Littermate IL-22^−/−^ or IL-22^+/+^ mice (*n* = 10) were assigned to receive either drinking water for 14 days followed by 2.5% DSS for 7 days and then drinking water again for 3 days. Prior to and during DSS treatment, groups were orally administered live Akk or PBS. The changes in body weight (**I**) and the disease activity index (**J**) were monitored daily following the DSS challenge. **K**, **L** Representative images of the colons from these mice (**K**), and measurement of colon length of the indicated mice (**L**). **M**–**O** Representative images of H&E stained (upper, scale bars 200 μm) and Alcian blue (below) stained colon sections from relevant groups (scale bars 100 μm). Histological scores of colitis (**N**), goblet cells in the crypt (**O**), and intestinal permeability (**P**) were quantitatively assessed. **Q**–**S** Representative images of immunohistochemistry (IHC) staining for Ki67 (upper) and immunofluorescence images of p-STAT3 staining (below) (**Q**) in the colon tissues from different treatment groups (scale bars 100 μm); The quantitative analysis of the number of ki67^+^ cells in the crypt (**R**) and the average median fluorescent intensity (MFI) of p-STAT3 (**S**) were assessed using ImageJ. *P < 0.05, **P < 0.01, ***P < 0.001, ****P < 0.0001

Gut microbiota significantly contributes to intestinal IL-22 production [6, 31]. We further extracted colonic lamina propria cells from healthy mice to verify in vitro the effect of Akk on the induction of IL-22 synthesis. Akk has demonstrated the potential to endure within immune cells in vitro [32]. Our results indicate that Akk remains viable in cell culture media, exhibiting a 1% survival rate after 24 h of oxygen exposure (Fig. S4A), which suggests a degree of oxygen tolerance and its potential applicability in short-term ex vivo co-culture experiments. Correspondingly, Akk stimulation increased IL-22 expression from ILC3s (Fig. 3A, B), despite stable ILC3s and Th cell numbers across treatments (Fig. S4B, C). Introducing TNF-α to simulate an inflammatory environment increased IL-22 secretion from Th cells, but not from ILC3s. Nevertheless, Akk further boosted IL-22 secretion by ILC3s under TNF-α stimulation, confirmed by ELISA assay of co-culture supernatants (Fig. 3C). Colonization of Akk aids epithelial development in germ-free mice [28]. Considering IL-22’s role in intestinal mucosa healing, we assessed Akk’s impact using an in vitro co-culture system of intestinal organoids and colonic LPLs. Unexpectedly, Akk alone didn't alleviate TNF-α-induced organoid damage, but its combination with LPLs improved organoid integrity (Fig. 3D, E and Fig. S4D). Immunofluorescence revealed p-STAT3 protein elevation and increased epithelial stem cell marker levels in the combined Akk-LPL treatment group (Fig. 3D, F-H). Interestingly, an anti-IL-22 antibody reversed Akk’s protective effect against TNF-α damage (Fig. 3D–H). Contrary to Akk’s in vitro promotion of IL-22 expression in LPLs, our in vivo results revealed that Akk supplementation did not significantly raise intestinal IL-22 levels in mice during homeostasis, potentially due to in vivo and in vitro differences (Fig. S4E). These findings suggest Akk induces IL-22 secretion from ILC3s, promoting intestinal mucosal repair under inflammation.

The role of IL-22 in Akk’s protective mechanism was further probed using IL-22 knockout (IL-22^−/−^) mice subjected to DSS-induced colitis. Remarkably, the absence of IL-22 amplified DSS-instigated intestinal inflammation, evidenced by significant weight loss, higher DAI and Histological scores, and reduced colon lengths. As hypothesized, the beneficial impact of Akk supplementation on wild-type mice's colitis was completely abolished in IL-22^−/−^ mice (Fig. 3I–N). Furthermore, no significant variations were found in goblet cell count and mucosal barrier permeability between DSS + Akk and DSS groups in these mice (Fig. 3M, O, P). Additionally, Akk treatment failed to salvage Ki67^+^ cells or p-STAT3 expression in these IL-22^–/–^ mice (Fig. 3Q–S). These notions underscore the pivotal role of IL-22 in Akk’s protective response towards DSS-induced colitis.

### Akk enhances RA synthesis of CD103^+^ DCs in mice with colitis

**Fig. 4:**
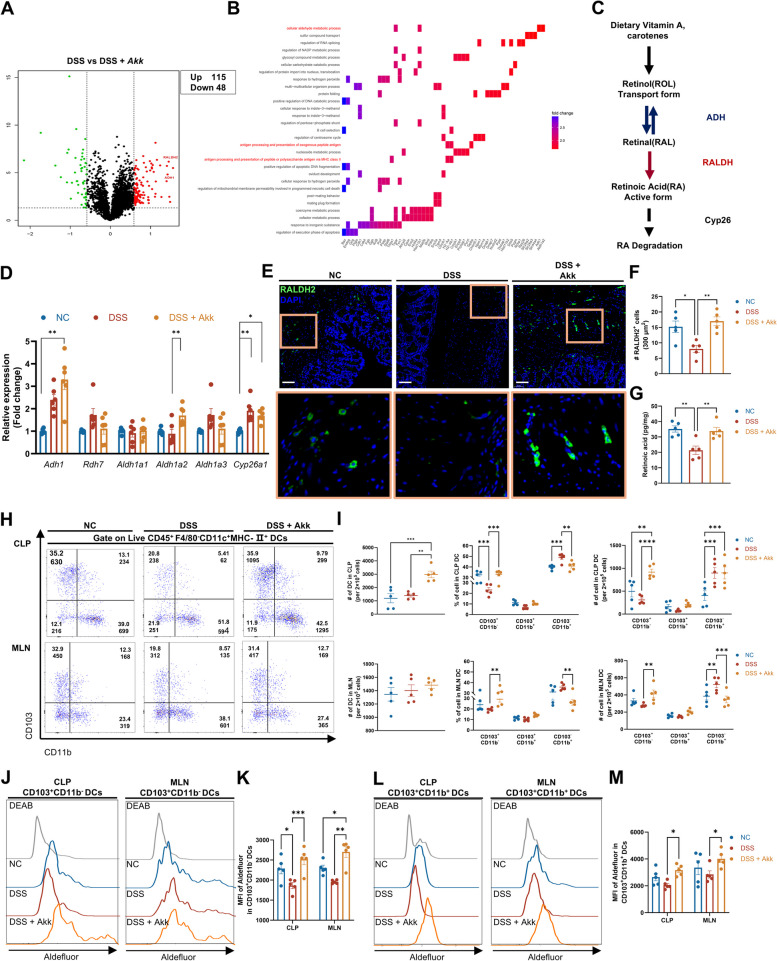
Akk promotes RA synthesis in CD103^+^ DCs in mice with colitis. **A**, **B** Volcano plot (**A**) and KEGG enrichment pathway analysis (**B**) display differentially expressed proteins and associated pathways among acute DSS-treated mice with gavage of PBS or Akk (*n* = 5). **C** Schematic diagram illustrating retinoic acid metabolism. Vitamin A undergoes two-step oxidation reactions mediated by retinol dehydrogenase (ADH) and retinal dehydrogenase (RALDH) to produce retinoic acid, which can further undergo degradation by cytochrome enzymes such as CY26A1. **D** Expression of retinoic acid metabolism-related genes was evaluated in bulk colon samples from untreated mice and acute DSS-treated mice with gavage of PBS or Akk (*n* = 6). **E**, **F** Representative immunofluorescence images of RALDH2 immunostaining (**E**) in colon tissues from relevant groups (scale bars 100 μm). Orange boxes represent zoomed-in views. The average number of RALDH2^+^ cells per 300 μm^2^ (**F**) was determined from 5 randomly selected fields of view (*n* = 5). **G** ELISA test of whole colon homogenates to evaluate retinoic acid production (*n* = 5). **H**, **I** Representative images (**H**), frequency, and quantitative analysis (**I**) of total and different DC subsets in the CLP (upper) and MLN (below) by flow cytometry (*n* = 5). **J**–**M** Representative images and quantitative analysis for FACS Aldefluor assay of CD103^+^CD11b^−^ DCs (**J**, **K**) and CD103^+^CD11b^+^ DCs (**L**, **M**) within the CLP and MLN (*n* = 5). *P < 0.05, **P < 0.01, ***P < 0.001

Utilizing iTRAQ proteomics analysis, we studied protein expression profiles of differing DSS-induced colitis treatment groups in mice. Akk treatment led to 115 upregulated and 48 downregulated proteins compared to DSS alone (Fig. 4A). Further exploration indicated a potential connection with the cellular RA metabolism pathway (Fig. 4B).

In vivo, RA synthesis occurs through a two-step process utilizing dietary vitamin A and retinol, catalyzed by alcohol dehydrogenase (ADH) and RALDH enzymes encoded by the *Adh* and *Aldh1a* gene families. Conversely, RA degradation primarily involves the enzyme cytochrome P45026A1 (CYP26A1) (Fig. 4C). Treatment with Akk resulted in a significant upregulation of *Adh1* and *Aldh1a2* mRNA expression, while the expression of *Aldh1a1*, *Aldh1a3*, and *Cyp26a1* remained unchanged (Fig. 4D). In comparison to the widespread presence of ADH, RALDH1 demonstrated a more restricted expression within intestinal epithelial cells (IECs), while RALDH2 was predominantly observed in immune cells, specifically DCs [33]. Immunofluorescence findings additionally confirmed the upregulation of RALDH2 expression and its localization in the lamina propria following Akk supplementation (Fig. 4E, F). The promotion of colonic RA synthesis by Akk supplementation was further substantiated through the usage of the ELISA test for detecting RA levels in the colon tissue homogenate (Fig. 4G).

The main source of RA, CD103^+^ DCs, including CD103^+^CD11b^−^ cDC1s and CD103^+^CD11b^+^ cDC2s, significantly declined in both quantity and function in cases of colitis [13, 14]. In contrast, CD103^−^CD11b^+^ cDC2s increase inflammatory conditions and promote TH17 immune responses [34]. As anticipated, flow cytometry revealed an augmentation in CLP DCs following Akk treatment. This was accompanied by an increment in CD103^+^CD11b^−^ cDC1s and a decrement in CD103^−^CD11b^+^ cDC2s in both CLP and MLN (Fig. 4H, I, Fig. S5A).

Consistent with previous research [13], the Aldefluor assay revealed higher RALDH activity in CD103^+^ compared to CD103^−^ DC subsets. Correspondingly, there was an increase in RALDH activity in CD103^+^ DC subsets of CLP and MLN (Fig. 4J–M, Fig. S5B, C). Following Akk treatment, an increased number of CLP and MLN macrophages was noted, but RALDH activity remained unchanged (Fig. S5D–G). In vitro experiments were conducted to confirm Akk’s ability to induce RALDH2 synthesis in DCs by applying Akk to isolated colonic LPLs from mice. Evidently, live Akk selectively enhanced RALDH activity in CD103^+^ DC subsets, without affecting the total number of DCs or their subset frequencies (Fig. S6A–E). However, this effect was nullified with heat-inactivated Akk. Additionally, infection levels or exposure times influenced RALDH activity in BMDCs due to Akk. Surprisingly, adding the immune adjuvant lipopolysaccharide (LPS) did not increase RALDH activity but decreased Akk's ability to trigger it (Fig. S6F–K).

Gut bacteria can produce RA independently of the host [17, 35]. We tested ALDH activity in different Akk strains and other gut bacteria, finding varying levels of activity (Fig. S7A). However, supplementing vitamin A did not increase RA levels in Akk cultures (Fig. S7B), suggesting Akk may not be able to synthesize RA in vitro. Interestingly, the short-term Akk supplementation in homeostatic mice partially elevates intestinal RA levels, emphasizing Akk’s key role in regulating host RA synthesis (Fig. S7C). Overall, our findings suggest that the quantitative and qualitative alterations in CD103^+^ DCs may form the basis for Akk’s capability to alleviate colitis in mice.

### RA synthesis in DCs is essential for Akk-induced IL-22 production in the inflamed colon

To study the role of DC-derived RALDH2 in Akk’s protective effect on colitis, we generated and utilized CD11c cre *Raldh2*^*fl/fl*^ (*Raldh2*^*ΔDC*^) mice. These mice exhibited reduced RALDH2 expression and activity in CD11c^+^ DCs, but not those in macrophages (Fig. S8A–E). These mice had fewer DCs in CLP, particularly CD103^+^CD11b^−^ DCs, as confirmed by Flowcytometry (Fig. S8F–I).

**Fig. 5:**
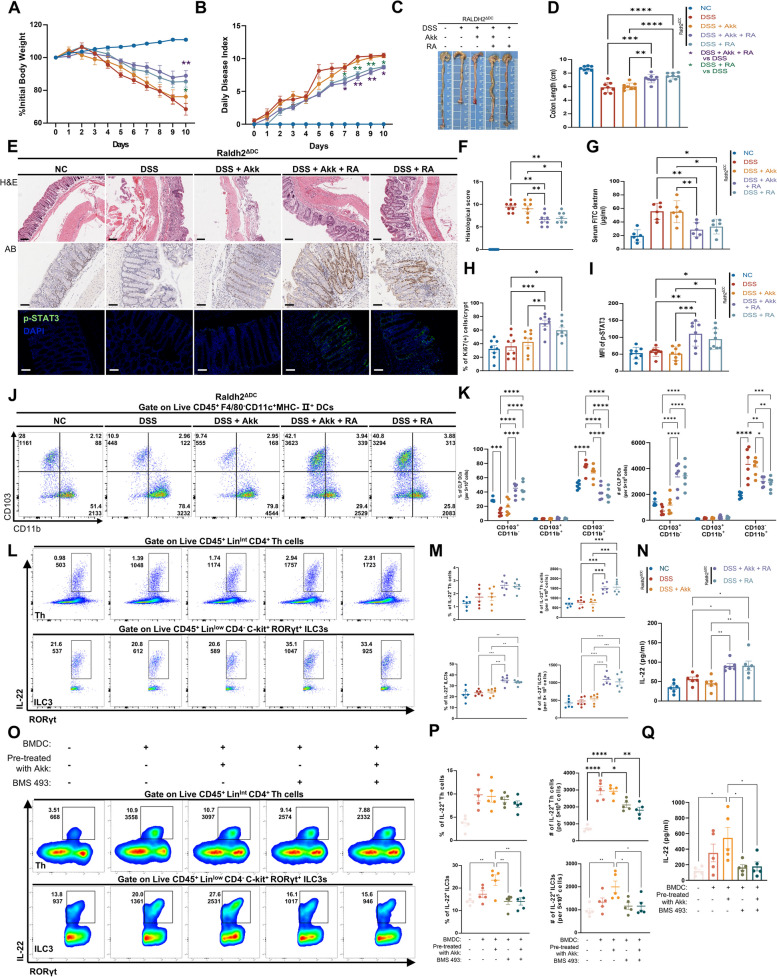
Retinoic acid synthesis in DCs is essential for Akk-induced IL-22 production in the inflamed colon. **A**–**D** The pathology of colitis in littermate *Raldh2*^*ΔDC*^ mice was assessed by observing untreated controls and test groups exposed to acute DSS with individual or combined treatments of Vehicle, Akk, or RA. Body weight changes (**A**), DAI (**B**), and colon lengths (C&D) were utilized to evaluate the pathology (*n* = 6). **E**, **F** Representative images of the H&E-stained(upper, scale bars 200 μm), the IHC images of Ki67 immunostaining colon sections (middle), and immunofluorescence images of p-STAT3 immunostaining (below) in the colon tissues of different treatment groups (scale bars 100 μm). Histological scores (**F**) were assessed in the indicated groups. **G** Intestinal permeability of the relevant groups was determined by FITC-dextran level in serum (*n* = 6). **H** Quantitative analysis of the percentage of ki67^+^ cells in the crypts by ImageJ (*n* = 6). **I** The average fluorescence intensity of p-STAT3 was quantitatively assessed from relevant groups (*n* = 6). **J**, **K** Representative images (**J**) and quantitative analysis (**K**) of different CLP DC subsets from various experimental groups were obtained through flow cytometry (*n* = 5). **L**, **M** Representative images (**L**) and quantitative analysis (**M**) of IL-22^+ ^Th cells and IL-22^+^ ILC3s in the CLP from various experimental groups were conducted via flow cytometry (*n* = 5). **N** The IL-22 production of the relevant groups was assessed by conducting ELISA tests on whole colon homogenates (*n* = 6). **O**–**Q** BMDCs, pre-treated with or without Akk, were then co-cultured with colonic LPLs, in the presence or absence of the RA pan-receptor inhibitor BMS493. Representative images (**O**), Representative flow plots (**O**), and both frequencies and quantitative analyses (**P**) of IL-22^+^ ILC3s and IL-22^+^ Th cells from relevant cells were conducted using flow cytometry. The IL-22 expression of co-cultured supernatants in relevant groups was determined by ELISA (**Q**) (*n* = 5). *P < 0.05, **P < 0.01, ***P < 0.001, ****P < 0.0001

By inducing colitis in *Raldh2*^*ΔDC*^ and littermate *Raldh2*^*fl/fl*^ mice via DSS, we found that Akk treatment resulted in mitigating DSS-induced colitis in wild-type (*Raldh2*^*fl/fl*^) mice as contrasted with the control group (Fig. S8J–O). In contrast, the deficiency of RALDH2 in DCs diminished Akk’s protective role against colitis as it failed to improve clinical parameters (Fig. 5A–D, Fig. S8M–P) or histological outcomes (Fig. 5E, F, Fig. S8Q, R). Moreover, Akk was not effective in either enhancing intestinal barrier permeability or activating p-STAT3 expression in *Raldh2*^*ΔDC*^ mice (Fig. 5E, G–I). Colitis in *Raldh2*^*ΔDC*^ mice was associated with an elevating CD11b^+^CD103^−^ DCs trend and a declining CD103^+^CD11b^−^ cDC1s trend from CLP, creating an imbalance unaddressed by Akk (Fig. 5J–K, Fig. S8 J). Noteworthy, the introduction of RA efficiently alleviated colitis in *Raldh2*^*ΔDC*^ mice and adeptly replenished the CD103^+^ DC population. Furthermore, the conjunct supplementation of RA and Akk markedly revitalized Akk’s anti-colitis effect in RALDH2^ΔDC^ mice (Fig. 5A–D), thus highlighting the key role of RA synthesis in DCs for Akk’s protective function against colitis.

The RA signal in intestinal DCs plays a crucial role in the secretion of IL-22 in ILC3s [12, 36]. Consistently, RALDH2 absence in DCs negates Akk’s augmentation of IL-22 secreting ILC3s, which can be restored with RA supplementation (Fig. 5L–N, Fig. S8K). Flow cytometric analysis shows Akk might boost ILC3s’ IL-22 secretion via promoting RA production in DCs, as examined in co-cultured colonic LPLs and Akk pre-treated BMDCs. Although the quantity of Th and ILC3s remained consistent (Fig. S8L), the addition of BMDCs slightly increased IL-22 secretion in ILC3s, which was further enhanced by Akk pre-treatment. BMDCs also caused a slight increase in IL-22 secretion in Th cells, with no additional effect from Akk. Notably, the pan-RA receptor inhibitor, BMS493, caused varying degrees of IL-22 secretion inhibition (Fig. 5O, P), validated through ELISA analysis of the co-culture supernatant (Fig. 5Q). Consistently, irrespective of the presence or absence of TNF-α, Akk is unable to effectively induce the secretion of IL-22 from the LPLs originating from *Raldh2*^*ΔDC*^ mice (Fig. S9A). In a co-culture system comprising LPLs from *Raldh2*^*ΔDC*^ mice and intestinal organoids, Akk alone was insufficient to mitigate TNF-induced damage or enhance p-STAT3 expression. Conversely, retinoic acid (RA) effectively attenuated the damage, and when administered in conjunction with Akk, it reinstated the protective effects of Akk (Fig. S9B–D).

To elucidate Akk’s contribution to the alleviation of colitis through the induction of RA synthesis, RA was orally administered to IL-22^-/-^ mice treated with DSS. The results showed that neither RA alone nor in combination with Akk had an impact on weight loss, disease activity index, or colon shortening in IL-22^-/-^ mice (Fig. S9E–J). This suggests that Akk’s promotion of RA synthesis primarily mitigates intestinal inflammation by enhancing IL-22 secretion by ILC3s.

### Akk promotes RA synthesis in DCs via the JAK2-STAT3 signaling pathway

**Fig. 6:**
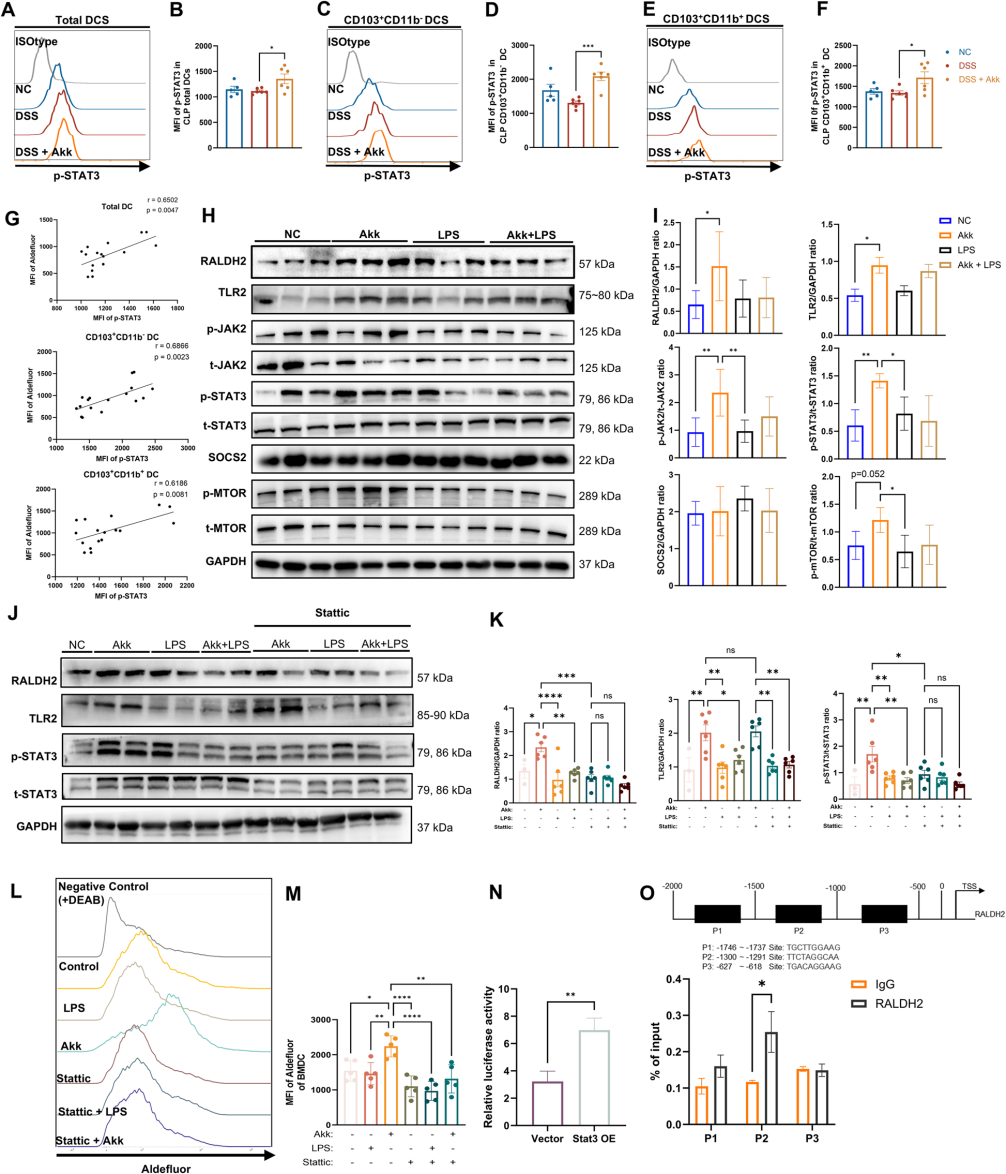
Akk promotes RA synthesis in DCs via the JAK2-STAT3 signaling pathway. **A**–**F** Representative images and quantitative analysis of Flow-cytometric analysis for p-STAT3 staining in the CLP total DCs (**A**, **B**), CD103^+^CD11b^−^ DCs (**C**, **D**), CD103^+^CD11b^+^ DCs (**E**, **F**) from untreated mice and acute DSS-treated mice with gavage of PBS or Akk (*n* = 5–6). **G** A correlation analysis was performed on total and CD103^+^ CLP DC subsets to determine the relationship between the MFI of p-STAT3 and Aldefluor (*n* = 17). **H**, **I** GM-CSF derived BMDCs were treated with LPS or Akk alone or together for 12 h. Representative immunoblot images of TLR2, p-JAK2, JAK2, p-STAT3, t-STAT3, SOCS2, p-mTOR, and mTOR in different treatment groups. Relative protein levels of indicated proteins were quantified to GAPDH. The protein levels of phosphorylated-STAT3 were quantified relative to total-STAT3 (*n* = 5). **J**, **K** GM-CSF derived BMDCs were treated with LPS or Akk alone or together, in the absence or presence of STAT3 inhibitor Stattic for 12 h. Representative immunoblot images of TLR2, p-STAT3, and t-STAT3 in different treatment groups. Relative protein levels for these proteins were calibrated to GAPDH while p-STAT3 levels were relative to total-STAT3 (*n* = 6). **L**, **M** Representative images (**L**) and quantitative analysis (**M**) of FACS Aldefluor assay for these indicated cells (*n* = 5). **N** The activity of the RALDH2 promoter was assessed by dual-luciferase reporter assay in BMDCs transfected with the vector or STAT3 overexpression (STAT3 OE) plasmids. **O** Schematic images of the potential STAT3 binding sites in the promoter of RALDH2 predicted by JASPAR (upper). ChIP analysis of STAT3 occupancy at the RALDH2 promoter in STAT3-overexpressing BMDCs (below). *P < 0.05, **P < 0.01, ***P < 0.001, ****P < 0.0001

STAT3 maintains immunosuppression in myeloid cells, including DCs [37, 38]. We hypothesized that Akk could regulate RA synthesis in DCs via STAT3 pathway activation. Flow cytometry analysis showed increased p-STAT3 expression in CLP DCs and CD103^+^ DC subsets after Akk treatment in colitis mice (Fig. 6A–F). Additionally, a strong correlation was seen between p-STAT3 levels and RALDH enzyme activity in these DC subsets (Fig. 6G), whereas STAT3 phosphorylation in CD103^−^ DCs and macrophages remained unaltered, with no significant link to RALDH enzyme activity (Fig. S10A–D).

Further analyses revealed Akk’s role in STAT3 signaling within BMDCs. Toll-like receptor 2 (TLR2) lacking may provoke DC stimulation by inhibiting the STAT3 pathway [39]. Akk elements, notably its outer membrane proteins and secreted intermediaries, can modulate immune responses via TLR2 [21, 23]. Consistently, Akk treatment significantly upregulated TLR2 expression in BMDCs. In addition, it amplified the phosphorylation of STAT3 and Janus-activated kinase 2 (JAK2) while Suppressor of cytokine signaling proteins 2 (SOCS2), the downstream inhibitory molecule, remains unaffected. Akk treatment also enhanced the phosphorylation levels of the mammalian target of rapamycin (mTOR), a known inducer of immunosuppression, either independently or in conjunction with STAT3 [40]. Contrarily, Lipopolysaccharides (LPS), a traditional immune-stimulating adjuvant derived from *E. coli*, failed to activate BMDC’s STAT3 pathway and inhibited Akk’s influence on both this path and RA synthesis in BMDC (Fig. 6H, I). Stattic [41], a specific STAT3 inhibitor, resulted in a significant decrease in both STAT3 and its phosphorylated forms within BMDCs, subsequently reducing the amplified RALDH2 expression (Fig. 6J, K), and activity (Fig. 6L, M) prompted by Akk.

Additionally, the regulatory effect of STAT3 on RALDH2 was explored by transfecting BMDCs with either empty vectors or STAT3 overexpression plasmids, resulting in a significant enhancement of RALDH2 expression due to STAT3 overexpression (Fig. S10E). STAT3 overexpression caused a significant increase in RALDH2 reporter activity, as evidenced by Luciferase reporter assays (Fig. 6N). The role of STAT3 in the transcriptional control of RALDH2 was further inspected. Utilizing the JASPAR database (https://jaspar.genereg.net/), three possible STAT3 binding sites were found in the RALDH2 promoter region (Fig. 6O, Fig. S10F). Chromatin immunoprecipitation followed by quantitative PCR (ChIP-qPCR) was conducted to verify these STAT3 binding sites, underlining the functional importance of the P2 site over P1 and P3 (Fig. 6O). Collectively, our results indicate the crucial reliance of Akk’s regulatory impact on RA synthesis in DCs on the activation of the STAT3 signaling pathway.

### Akks exhibit strain-specific effects in ameliorating acute colitis in mice

**Fig. 7:**
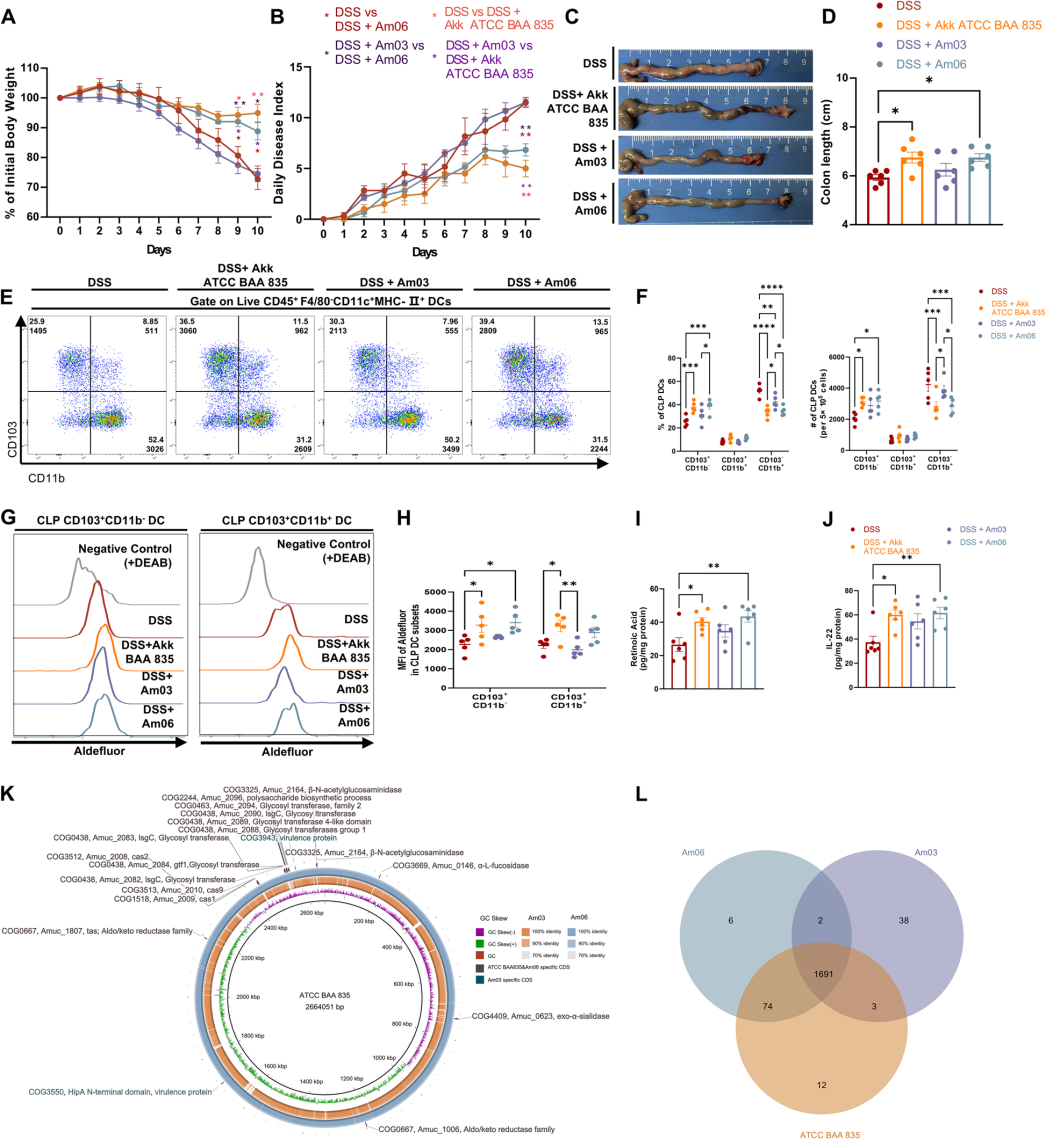
Akk exhibits strain-specific effects in ameliorating acute colitis in mice. **A**–**D** The pathology of colitis was evaluated among the 4 acute colitis groups with PBS, Akk ATCC BAA-835, Am03, or Am06 gavage respectively. Body weight changes (**A**), DAI (**B**), and colon lengths (**C**, **D**) were utilized to evaluate the pathology (*n* = 6). **E**, **F** Representative images (**E**), frequencies, and quantitative analysis (**F**) of different CLP DC subsets from relevant groups were assessed by Flow cytometry (*n* = 5). **G**, **H** Representative images (**G**) and quantitative analysis (**H**) for FACS Aldefluor assay of CLP CD103^+^CD11b^−^ DCs and CD103^+^CD11b^+^ DCs from relevant groups (*n* = 5). **I**, **J** ELISA test of whole colon homogenates to evaluate RA (**I**) and IL-22 (**J**) production from relevant groups (*n* = 6). **K** Genomic comparison of Akk ATCC BAA-835, Am03, or Am06 chromosomes, starting from the predicted replication origin, includes (1) Am03 or Am06 contigs, (2) ATCC BAA-835 genome identity based on BLASTN against Am03 or Am06, and (3) interested strain-specific coding sequences (CDSs) for Am03, along with unique CDSs for ATCC BAA-835 and Am06, with annotated functions. **L** Visualization of the Eggnog output comparing the number of unique and shared orthologs of Akk BAA 835, Am03, and Am06. *P < 0.05, **P < 0.01, ***P < 0.001, ****P < 0.0001

We evaluated if different Akk strains could alleviate murine colitis by promoting RA synthesis in DCs. Comparing Akk strains Am03 and Am06, derived from healthy donors’ feces or breast milk [42], to the type strain ATCC BAA-835, it was evident that Am06 had effects similar to ATCC BAA-835 in treating acute colitis, while Am03 showed no significant therapeutic benefits (Fig. 7A–D, Fig. S11A, B). In contrast, Am03 led to persistent goblet cell loss and increased intestinal permeability, like the DSS group (Fig. S11A–C, E). Unlike ATCC BAA-835 or Am06, Am03 did not counteract the decrease in crypt Ki67-positive cells and p-STAT3 induced by colitis (Fig. S11D, F, G). Flow cytometry confirmed that Am06, like ATCC BAA-835, restored CD103^+^CD11b^−^ cDC1s and RALDH enzyme activity (Fig. 7E–H). We evaluated if different Akk strains could alleviate murine colitis by promoting RA synthesis in DCs. Comparing Akk strains Am03 and Am06, derived from healthy donors’ feces or breast milk [42], to the reference strain ATCC BAA-835, it was evident that Am06 had effects similar to ATCC BAA-835 in treating acute colitis, while Am03 showed no significant therapeutic benefits (Fig. 7A–D, Fig. S11A, B). In contrast, Am03 led to persistent goblet cell loss and increased intestinal permeability, like the DSS group (Fig. S11A–C, E). Unlike ATCC BAA-835 or Am06, Am03 did not counteract the decrease in crypt Ki67-positive cells and p-STAT3 induced by colitis (Fig. S11D, F, G). Flow cytometry confirmed that Am06, like ATCC BAA-835, restored CD103^+^CD11b^−^ cDC1s and RALDH enzyme activity (Fig. 7E–H). Accordingly, despite the observation of a minor upward trend, Am03 did not prove as effective as either Am06 or ATCC BAA-835 in augmenting the levels of RA and IL-22 in the murine colon (Fig. 7I, J). To determine the relationship between the colonization of different Akk strains and their therapeutic effects, qPCR and FISH assays were carried out. Surprisingly, no significant differences in Akk abundance were found in the colon mucosa across different Akk strains treatments (Fig. S11H, I), indicating extrinsic factors might be accountable for the differential therapeutic effectiveness observed between Akk strains. The eggNOG analysis of Clusters of Orthologous Groups of proteins (COGs) reveals no significant differences across all core functional clusters within the genomes of three Akk strains (Fig. S10G). However, comparative genomic analysis indicates that Am03 harbors the most strain-specific genes, including two putative virulence factor clusters: COG3550 and COG3943 [43, 44] (Fig. 7K, L, Table S3). Conversely, Am06 displays a larger quantity of strain-specific genes that are present in Akk reference strains. Furthermore, when compared to Am03, both Am06 and Akk reference strains can encode a wider variety of sialidases (*Amuc_0146*) and fructosidases (*Amuc_0623*), suggesting superior adaptation to the mucin interface and a differential preference for metabolic substrates [45]. Overall, these results suggest that the therapeutic effect of Akk on colitis may be strain-specific. Am06 displays a consistent capability for inducing RA synthesis and IL-22 secretion, much like the Akk BAA-835 strains, further bolstering the significant role of RA synthesis in reducing colitis by Akk.

## Discussion

The gut microbiota is important in UC development, but the specific role of different strains is unclear. In our mouse model, by activating the JAK2-STAT3 signaling pathway, live Akk supplementation increases CD103^+^ DCs in the gut and promotes their synthesis of RA. Subsequently, this leads to the secretion of IL-22 by ILC3s and mitigates colitis. However, heat inactivation may reduce the effectiveness of Akk treatment. Our findings elucidate the novel Akk/RA/IL-22 axis as a regulatory pathway in the progression of UC. Furthermore, the novel Akk strain Am06 isolated from breast milk shows consistent RA synthesis and IL-22 induction abilities, like the reference strain, and thereby effectively protects against experimental colitis.

Pasteurized Akk demonstrates therapeutic potential in metabolic diseases [21, 22], yet its viability has also been noted as crucial [24, 46]. Our research supports this finding, in that live Akk demonstrates efficient colonization in the colon and concomitant alleviation of colitis. However, heat-inactivated Akk or lower Akk dosages exhibit no such efficacy, highlighting the crucial role of Akk’s survival status. While heat inactivation resulted in the loss of preventive effects of Akk on the mouse sepsis model [24], secreting protein P9 by Akk has shown better metabolic regulation than its outer membrane protein *Amuc_1100* [47]. This underlines the predominant effect of Akk’s bioactive secreted substances in its therapeutic function. However, we found that the therapeutic effects of Akk CM supernatant vary from live Akk, likely due to in vivo and in vitro environmental differences and culture medium confounders. Variations in growth and metabolism were detected in Akk cultures with or without mucin [48], the latter showcasing superior mucosal barrier enhancement [49]. These findings align with our observations, highlighting the efficacy of mucin-free cultured Akk in alleviating colitis. Our recent work achieved robust Akk growth in a defined culture system, allowing an assessment of safety and addressing metabolic issues from complex animal-origin culture components [42]. An additional study is required to evaluate the metabolic and probiotic properties of Akk strains within this system. Future research should prioritize metabolomics analysis and use germ-free mice to pinpoint key bioactive metabolites produced by Akk.

While RA’s link to the immune system is well-established, we've only recently understood the gut microbiome’s role in RA metabolism [7, 17]. Vitamin A deficiency can alter mouse gut microbiota [50], while disruptions in the microbiota can hinder RA synthesis and worsen colitis-associated colorectal cancer [51]. Conversely, certain gut bacteria can produce RA internally, boosting early protection against pathogens [17]. Gut bacteria may differentially manipulate RA metabolism in the host. Interestingly, despite the presumed presence of the ALDH protein (ACD05876.1), We failed to find RA directly synthesized by Akk, possibly due to substrate preference and catalytic activity. In contrast, our data elucidate a novel mechanism where Akk restores diminished RA synthesis in CLP CD103^+^ DCs, thereby elevating RA levels through a host-dependent pathway and alleviating colitis in mice. Additionally, Akk predominantly boosts RA synthesis via RALDH2 in DCs, not affecting RA synthase activity in other cell types such as intestinal macrophages or IECs that mainly express RALDH1 [14, 52, 53]. *Lactobacillus intestinalis* has similar effects on colonic RA levels without impacting RALDH1, contrasting with other ALDH-active bacteria like *Escherichia coli* [35], emphasizing the unique impacts of symbiotic bacteria on immunity. Notably, different RALDH isoenzymes exhibit unique expression patterns and developmental roles, suggesting their specificity [54]. While limitations are present in conducting detailed assessments of individual RALDH isoenzyme contributions to RA metabolism, new techniques offer potential insights [55], particularly on Akk's role through differential RALDH isoenzyme regulation. Prior studies have illustrated that various active forms of Akk trigger markedly different transcriptional responses within host cells [32, 56]. Our in vitro findings support this, suggesting that live Akk, unlike its inactivated counterpart, effectively induces RALDH2 expression in DCs. This implies that the impact of Akk on RA synthesis is primarily driven by Akk's activity rather than bacterial contact. Extant literature establishes that microorganisms can reside within gastrointestinal immune cells, thereby aiding immune regulation [57]. In line with this, Ainize et al. demonstrate that live Akk can temporarily colonize and survive within macrophages in vitro [32]. Aligning with this finding, our FISH results show that colonic lamina propria cells further engulf Akk when administered live Akk, a response not seen in the heat-inactivated Akk group. These collective findings emphasize Akk’s potential to inhibit immune cells and carry out immune regulatory functions. However, although Am03 can also be engulfed by colonic lamina propria cells, it did not effectively induce RA synthesis in DCs. Reemphasizing the importance of earlier discussions, metabolic studies of Akk, conducted based on a defined culture system, are crucial to further elucidate Akk’s primary bioactive effector. Regardless of the exact underlying mechanisms, our robust data strongly supports the paramount role of DC-mediated RA synthesis in mitigating Akk-involved mouse colitis.

RA is important for maintaining intestinal balance by promoting Treg differentiation [7], but can also trigger Th17 response in certain situations [58]. Akk supplementation can restore Tregs and IL-10 levels but may not impact IL-17 levels in colitis mice, highlighting the intricate nature of the inflammatory environment. Moreover, RA-mediated IL-22 production by ILC3s is crucial for mucosal integrity and healing [12, 59], with microbiota playing a significant role. By regulating RA synthesis in IECs, commensal *clostridia* diminish IL-22 production and the subsequent anti-microbial responses [31]. In contrast, *B. fragilis* strain ZY-312 can alleviate colitis in an IL-22-dependent manner [6]. Consistent with recent research [60], our results reveal a marked increase in ILC3 numbers and IL-22 production post-Akk treatment. By using IL-22 knockout mice, we confirmed IL-22’s role in the anti-inflammatory effects initiated by Akk. ILC3s depend more on stimulation from LPLs than a direct response to intestinal bacteria for IL-22 production [61]. We consistently found that Akk-pretreated BMDCs promote IL-22 secretion by ILC3s, which is inhibited by pan-RA receptor inhibitors. The lack of RALDH2 in DCs negates the increase of IL-22^+^ ILC3s caused by Akk, highlighting RA’s importance in IL-22 production post-Akk exposure. Consistently, the inability to alleviate colitis in IL-22^-/-^ mice with RA alone or combined with Akk confirms IL-22’s essential role in RA-related mucosal repair. Our work shows a novel interaction between gut microbiota and host immunity and the importance of RA-induced IL-22 in protecting against colitis by Akk.

While human-derived Akks show high similarity in the 16S rRNA sequence, variations occur in host preferences and functional features among identified subspecies [62]. Breast milk is critical in defining an infant's early gut microbiota [63]. Our past research indicates that the breast milk-derived Am06 strain surpasses fecal-derived Akk in preventing certain diseases [64]. Supporting this, our current study shows that Am06 alleviates colitis and enhances RA synthesis in mice, similar to the Akk reference strain. However, these effects are not mirrored by the fecal-derived strain Am03, implying a strain-specific variation. Our previous studies have found metabolic similarities between Am06 and the reference strain, with an Average Nucleotide Identity (ANI) of 99.99%, compared to 97.31% for Am03 [42]. Genomic analysis shows that different strains of Akk bacteria have specific genes that may explain their therapeutic differences. Akk’s sialidases and fucosidases could help it thrive in mucus [45]. The genomes of Am06 and the Akk reference strain contain more of these enzyme genes, potentially facilitating Akk’s colonization and survival. In contrast, Am03 exclusively harbors two potential virulence genes. Bacterial colonization may increase glycosyltransferase levels in mice intestines [65], with disorderly glycosylation patterns apparent in human diseases [66]. Several glycosyltransferases, such as *Amuc_2087-2090* and *Amuc_2093-2097*, are unique to AM06 and ATCC BAA-835. Studies note a correlation between Akk abundance in patients’ gastrointestinal tracts and response to immune checkpoint inhibitors (ICI) [67], with different structural variants of Akk’s glycosyltransferases potentially impacting therapy outcomes in melanoma patients [68]. While these studies highlight the importance of certain genes in bacterial functions, more validation is necessary. New studies propose a gene editing technique for Akk [69], which could clarify specific genes’ roles in immunoregulatory actions.

## Conclusion

Our study illuminates the intricate relationship between the gut microbiome and immune mechanisms in UC and highlights Akk’s therapeutic promise. We found that live Akk significantly reduces colitis severity in a DSS-induced mouse model, an effect not replicated by heat-killed or pasteurized Akk. Based on our findings, we propose a hypothesis for the effect of the Akk supplement on UC-like colitis (Fig. S11). Exogenous supplementation of decreased Akk in UC may contribute to the restoration and functional improvement of CD103 + DCs. This, in turn, amplifies retinoic acid synthesis through the JAK2-STAT3 pathway and RALDH2 enzyme expression. Moreover, it escalates the production of IL-22 by ILC3s, thereby enhancing the integrity of the colonic mucosal barrier and facilitating mucosal repair in DSS-induced colitis. Notably, different Akk strains exhibit varying efficacies, with the human breast milk-derived strain Am06 showing consistent effectiveness. These findings suggest that a particular Akk strain could offer new strategies for UC treatment by modulating specific immune pathways.

## Supplementary Information

Supplementary Material 1.

## Data Availability

All data relevant to the study are included in the article or uploaded as online supplemental information. The proteomics data reported in this paper have been deposited in the ProteomeXchange Consortium via the iProX partner repository (accession No. PXD052243). The sequence data were submitted to the SRA database under accession number PRJNA915585.

## Abbreviations

UC: Ulcerative colitis
RA: Retinoic acid
ROL: Retinaldehyde
DAI: Disease Activity Index
MFI: Mean fluorescence intensity
DEAB: Diethylaminobenzaldehyde
Akk: *Akkermansia muciniphila*
DCs: Dendritic cells
ILC3s: Group 3 innate lymphoid cells
Tregs: Regulatory T cells
IECs: Intestinal epithelial cells
LPLs: Lamina propria lymphocytes
CLP: Colonic lamina propria
MLN: Mesenteric lymph nodes
LPS: Lipopolysaccharide
RALDH2: Retinaldehyde dehydrogenase 2
STAT3: Signal transducer and activator of transcription 3
Lgr5: Leucine-rich repeat-containing G-protein coupled receptor 5
Ascl2: Achaete-scute family bHLH transcription factor 2
ADH: Alcohol dehydrogenase
TLR2: Toll-like receptor 2
JAK2: Janus kinase 2
SOCS2: Suppressor of cytokine signaling 2
mTOR: The mechanistic target of rapamycin
SFB: Segmented filamentous bacteria
ANI: Average Nucleotide Identity
COG: Clusters of Orthologous Groups
ICI: Immune checkpoint inhibitors
