# Correction: Live *Akkermansia muciniphila* boosts dendritic cell retinoic acid synthesis to modulate IL-22 activity and mitigate colitis in mice

**DOI:** 10.1186/s40168-025-02060-7

**Published:** 2025-02-19

**Authors:** Hongbin Liu, Ruo Huang, Binhai Shen, Chongyang Huang, Qian Zhou, Jiahui Xu, Shengbo Chen, Xinlong Lin, Jun Wang, Xinmei Zhao, Yandong Guo, Xiuyun Ai, Yangyang Liu, Ye Wang, Wendi Zhang, Fachao Zhi

**Affiliations:** 1 Department of Gastroenterology, Guangdong Provincial Key Laboratory of Gastroenterology, Institute of Gastroenterology of Guangdong Province, Nanfang Hospital, Southern Medical University, Guangzhou, China; 2 Department of Gastroenterology, The Second Affiliated Hospital of Guangzhou, University of Chinese Medicine, Guangzhou, China; 3 Department of Gastroenterology, The Second Affiliated Hospital of Guangzhou Medical University, Guangzhou, China; 4 Department of Gastroenterology, Institute of Digestive Diseases, The Affiliated Qingyuan Hospital (Qingyuan People’s Hospital), Guangzhou Medical University, Qingyuan, China; 5 Huiqiao Medical Center, Nanfang Hospital, Southern Medical University, Guangzhou, China; 6 Guangzhou ZhiYi Biotechnology Co., Ltd, Guangzhou, China

**Correction: Microbiome 12, 275 (2024)**

**https://doi.org/10.1186/s40168-024-01995-7**

Following publication of the original article [1], the author reported that Supplementary files 1 and 2 should be removed and Supplementary 3 should now be Supplementary 1.

The original article has been updated.
