# Supplementary material for: Live Akkermansia muciniphila boosts dendritic cell retinoic acid synthesis to modulate IL-22 activity and mitigate colitis in mice
Source: Microbiome. 2024 Dec 30;12:275. doi: 10.1186/s40168-024-01995-7 (PMC11684322; doi:10.1186/s40168-024-01995-7)
Supplement: Supplementary file 2 — Supplementary Material 1. [file 40168_2024_1995_MOESM1_ESM.docx]

**Supplementary Information**

**Live *Akkermansia muciniphila* Boosts Dendritic Cell Retinoic Acid Synthesis to Modulate IL-22 Activity and Mitigate Colitis in Mice**

**Hongbin Liu** ^1, †^, **Ruo Huang**^1, †^, **Binhai Shen** ^1, †^, **Chongyang Huang** ^2^, **Qian zhou**^1^, **Jiahui Xu**^3^, **Shengbo Chen** ^4^, **Xinlong Lin**^1^**,** **Jun Wang** ^2^, **Xinmei Zhao**^1^, **Yandong Guo**^1^, **Xiuyun Ai**^5^, **Yangyang Liu** ^6^, **Ye Wang**^6^, **Wendi Zhang**^1, *^, **Fachao Zhi**^1, *^

^1^Guangdong Provincial Key Laboratory of Gastroenterology, Institute of Gastroenterology of Guangdong Province, Department of Gastroenterology, Nanfang Hospital, Southern Medical University, Guangzhou, China

^2^Department of Gastroenterology, The Second Affiliated Hospital of Guangzhou University of Chinese Medicine, Guangzhou, China

^3^ Department of Gastroenterology, The Second Affiliated Hospital of Guangzhou Medical University, Guangzhou, China

^4^ Department of Gastroenterology, Institute of Digestive Diseases, the Affiliated Qingyuan Hospital (Qingyuan People's Hospital), Guangzhou Medical University, Qingyuan, China

^5^ Huiqiao Medical Center, Nanfang Hospital, Southern Medical University, Guangzhou, China

^6^Guangzhou ZhiYi Biotechnology Co., Ltd., Guangzhou, China

^†^These authors contributed equally to this study

^*^Correspondence and requests for materials should be addressed to W.Z. (zhang_wendi@163.com) and F.Z. (zhifc41532@163.com)

**Supplementary Materials and Methods**

**1. Histological analysis, immunohistochemistry, and immunofluorescence detection**

Mouse colon tissue was fixed with 4% paraformaldehyde, embedded in paraffin, and sectioned into 4µm thick slices. Following hematoxylin and eosin (H&E) staining, the sections were evaluated and scored in a blind manner, adhering to predetermined standards. Alcian blue staining was conducted using a Biossci (China) protocol. For immunohistochemistry, the colon sections were subjected to deparaffinization, hydration, antigen retrieval, endogenous peroxidase quenching, and blocking. Afterwards, the sections were subjected to overnight incubation at 4°C with primary antibodies against Ki67, RALDH2, or p-STAT3, followed by 30 minutes of incubation with biotinylated secondary antibodies. Visualization was facilitated using a 3,3'-Diaminobenzidine Kit (ZSGB-BIO, China). Immunofluorescence staining was carried out on the colon sections similarly and utilized FITC-conjugated goat anti-rabbit IgG antibodies (Beyotime, China) for indirect immunofluorescence staining.

Colon organoids were fixed with paraformaldehyde and incubated overnight at 4°C with anti-p-STAT3 antibodies to conduct immunofluorescent staining. Subsequently, these organoids were incubated for an hour at ambient temperature with FITC-conjugated goat anti-rabbit IgG (Beyotime, China), and then imaged under an Olympus IX73(Japan) fluorescence microscope.

**2. Measurement of intestinal barrier permeability**

The assessment of intestinal barrier permeability in mice followed protocols established in prior studies[1]. In summary, FITC-dextran (Sigma, US) was orally administered to mice 4 hours before euthanasia. The fluorescence of FITC-dextran in the serum was then measured using a SpectraMax M3 multifunctional microplate reader (Molecular Devices, US), with an excitation wavelength of 485 nm and an emission wavelength of 528 nm.

**3. Proteomic Analysis**

We leveraged iTRAQ technology to discern differentially expressed proteins in colon samples from untreated mice compared to acute DSS-treated mice receiving PBS or Akk gavage. Proteins showing significant difference were identified using a False Discovery Rate (FDR) of <0.05 and a fold change ratios of ≥ 1.5 or ≤ 0.67 as cut-offs. We conducted a volcano plot analysis and performed Gene Ontology (GO) and Kyoto Encyclopedia of Genes and Genomes (KEGG) enrichment analyses on these proteins, using the ClusterProfiler R package[2]. Protein sequencing and analysis were executed by Novogene Co., Ltd. (Beijing, China).

**4. Enzyme-linked immunosorbent assay**

Colon tissue samples from mice were homogenized in phosphate-buffered saline (PBS) with a protease inhibitor (Beyotime, Shanghai, China), ensuring light exposure was minimized during the process. Total protein concentrations were quantified using a bicinchoninic acid assay. As per the manufacturers' guidelines, numerous ELISA kits were deployed to assess the levels of cytokines inclusive of TNF-α, IL-6, IL-10, IL-12, IL-17, IL-22, IL-23, IFN-γ, as well as the retinoic acid level, in the colon tissue of the mice.

**5. Western blot**

We conducted Western blotting following previously outlined methods[3]. The proteins were sorted using SDS-PAGE and subjected to immunoblotting analysis with rabbit polyclonal antiserum against p-STAT3 (1:1000), STAT3 (1:1000), p-JAK2 (1:1000), JAK2 (1:1000), SOCS2 (1:1000), TLR2 (1:800), p-mTOR (1:1000), mTOR (1:1000), and RALDH2 (1:1000). GAPDH (1:3000) antibodies were employed to quantify colonic epithelial protein expression.

**6. RNA isolation and real-time PCR**

Total RNA from colonic tissue and organoids was extracted using a column-based isolation kit (EZbioscience, Roseville, MN, USA), following the manufacturer’s instructions. RNA concentrations were determined using NanoDrop 2000 (Thermo Fisher, Waltham, MA, USA). Complementary DNA (cDNA) was synthesized using reverse transcription kits (Accurate Biology, Guangzhou, China). Primer details are provided in the Table S4.

**7. Bone marrow-derived dendritic cells (BMDCs) isolation and differentiation**

Under sterile conditions, the tibias and femurs of mice were carefully cleaned of adherent tissues. The bones were incised at the midsection to expose the medullary cavity, which was then thoroughly flushed with ice-cold PBS solution until it ran clear. The resulting fluid was collected through a 70μm cell strainer and centrifuged, after which the supernatant was discarded. The cell pellet, post-red blood cell lysis, was resuspended in IMDM culture medium (Gibco, US) supplemented with 10% FBS (Gibco, US), 25 mM HEPES (Gibco, US), 1% non-essential amino acids (Gibco, US), 1% sodium pyruvate (Gibco, US), and 1% penicillin-streptomycin solution (Gibco, US). Depending on the exact experimental needs, GM-CSF (Peprotech, US) was added to a final concentration of 20 ng/mL, or FLT3L (Peprotech, US) was included to reach a final concentration of 200 ng/mL. The BMDCs were then cultured at 37°C in an atmosphere containing 5% CO_2_, with half of the medium refreshed every 3 days. At the end of a 7-day culture period, the BMDCs were harvested by softly agitating the culture dish and were ready for subsequent analysis.

**8. ALDH activity assay**

Bacterial pellet samples were homogenized in ice-cold ALDH assay buffer and centrifuged at 4°C at maximum speed for 10 minutes. The supernatant concentrations were then determined using a bicinchoninic acid assay. Following this, an ALDH activity assay was conducted in accordance with the manufacturer's guidelines. This involved mixing equal volumes of protein-normalized supernatants with ALDH substrate and acetaldehyde, followed by incubation at room temperature. Absorbance at 450 nm was measured at 3-minute intervals over 30 minutes using a SpectraMax M3 multifunctional microplate reader (Molecular Devices, US). ALDH enzyme activity was quantified based on the production of NADH.

**9. Dual Luciferase Reporter Assay**

In this study, we utilized a variety of plasmids from GenePharma (Jiangsu, China), including the Aldh1a2 promoter reporter plasmid, pRL-CMV Renilla luciferase reporter plasmid, mouse STAT3 overexpression plasmid [pcDNA3.1(+) STAT3], and its corresponding negative control [pcDNA3.1(+)]. The Aldh1a2 promoter reporter plasmid was constructed by amplifying the Aldh1a2 upstream region (-2000 to +100 bp) from mouse genomic DNA via PCR and inserting this fragment upstream of the firefly luciferase construct in the pGL4.10 vector. Similarly, the full-length cDNA of mouse STAT3 was cloned into the pcDNA3.1 vector to create the pcDNA3.1(+) STAT3 plasmid. For the luciferase assay, BMDCs were transfected with either the mouse STAT3 overexpression plasmid [pcDNA3.1(+) STAT3] or its negative control [pcDNA3.1(+)], along with the Aldh1a2 promoter reporter plasmid and pRL-CMV Renilla luciferase reporter plasmid using Zeta life Advanced DNA RNA Transfection Reagent (CA, USA). After 48 hours of incubation, luciferase assays were performed using the Beyotime Dual Luciferase Reporter Assay kit (Shanghai, China), with Renilla luciferase activity serving as the internal control.

**10. chromatin immunoprecipitation (ChIP) assay**

The pcDNA3.1(+) STAT3 plasmid was transfected into BMDCs. Following 48 hours, a chromatin immunoprecipitation (ChIP) assay was executed utilizing the SimpleChIP Enzymatic Chromatin IP Kit (CST, CA). Briefly, 40 million BMDC cells were subjected to crosslinking with formaldehyde, followed by digestion and sonication to yield suitably fragmented genomic DNA. Subsequently, immunoprecipitation was performed using either the anti-Stat3 antibody or the control rabbit IgG. The purified DNA samples were then quantified via RT-PCR. The Aldh1a2 gene's upstream genomic DNA sequence, spanning 2 kb from the transcription start site, was extracted from the Genome Browser (University of California, Santa Cruz, Santa Cruz, CA). Gene-specific primers for the ChIP experiment were devised at three positions: site 1 (-1850 ~ -1650bp), site 2 (-1400 ~ -1200bp), and site 3 (-750 ~ -550bp). The final data is expressed as the percentage of DNA, bound by the target transcription factor, normalized to the total input DNA.

**11. Colonic crypt isolation and colonic organoid co-culture construction**

The colons of 4-week-old C57BL/6 mice were isolated, washed, and fragmented. After thorough washing with PBS, the fragments were immersed in Gentle Cell Dissociation Reagent (Stem Cell, CA) and incubated on a shaking platform for 20 minutes. The resulting precipitate was filtered through a 70 μm cell strainer, followed by additional washing with DMEM/F12 supplemented with 20 mM HEPES. The purified colonic crypts were obtained through centrifugation and counted under a microscope. 500 colonic crypts per well were suspended in equal amounts of IntestiCult organoid growth medium (Stem cell, CA) and Matrigel (Corning, US). Subsequently, 50 μL of the mixture was added to the center of each well of a 24-well plate. After gel solidification, additional medium was added. To investigate the role of cytokine, on the seventh day, colonic lamina propria lymphocytes(LPLs) were isolated as described above and added to the culture medium at a cell ratio of 10^5^ cells per well for indirect co-culture. The organoids were co-cultured with or without AKK (10^4^ CFU/well) and/or mouse TNF-α (50 ng/mL; Peprotech, US), and/or treated with a neutralizing antibody against mouse IL-22 (0.1 μg/mL, RD, US) for 24 hours.

**12. Relative Quantification of *A. muciniphila* by q-PCR**

A longitudinal incision was made in a mouse’s colon and the contents were gently removed. The mucus layer was then collected with a sterile cotton swab to analyze the relative abundance of *A. muciniphila* in the colon mucosa. The extraction of bacterial DNA was conducted using the TIANamp Stool DNA Kit (TIANGEN, Beijing, China). Finally, q-PCR was utilized to evaluate the quantity of A. muciniphila, adhering to methodologies reported in past research[4]. Primer details are provided in the Table S4.

**13. Fluorescent In situ Hybridization (FISH)**

The colon tissue slices, which contained fecal material, were cleaned and treated with lysozyme prior to undergoing FISH, as per the standard protocol[4,5]. The Akkermansia-specific probe sequence employed was CCTTGCGGTTGG-CTTCAGAT, which was tagged at the 5’end with Cy5. Finally, the slides were prepared with the application of DAPI-containing antifade mounting media (Beyotime) and then imaged under an Olympus IX73(Japan) fluorescence microscope.

**Reference**

1. He C, Deng J, Hu X, Zhou S, Wu J, Xiao D, et al. Vitamin A inhibits the action of LPS on the intestinal epithelial barrier function and tight junction proteins. Food Funct. 2019;10:1235–42.

2. Wu T, Hu E, Xu S, Chen M, Guo P, Dai Z, et al. clusterProfiler 4.0: A universal enrichment tool for interpreting omics data. The Innovation [Internet]. 2021 [cited 2024 Dec 7];2. Available from: https://www.cell.com/the-innovation/abstract/S2666-6758(21)00066-7

3. Zhang W, Zhou Q, Liu H, Xu J, Huang R, Shen B, et al. Bacteroides fragilis strain ZY-312 facilitates colonic mucosa regeneration in colitis via motivating STAT3 signaling pathway induced by IL-22 from ILC3 secretion. Front Immunol. 2023;14:1156762.

4. Alam A, Leoni G, Quiros M, Wu H, Desai C, Nishio H, et al. The microenvironment of injured murine gut elicits a local pro-restitutive microbiota. Nat Microbiol. 2016;1:15021.

5. Everard A, Belzer C, Geurts L, Ouwerkerk JP, Druart C, Bindels LB, et al. Cross-talk between Akkermansia muciniphila and intestinal epithelium controls diet-induced obesity. Proc Natl Acad Sci U S A. 2013;110:9066–71.

**Supplementary Figure S1-12**


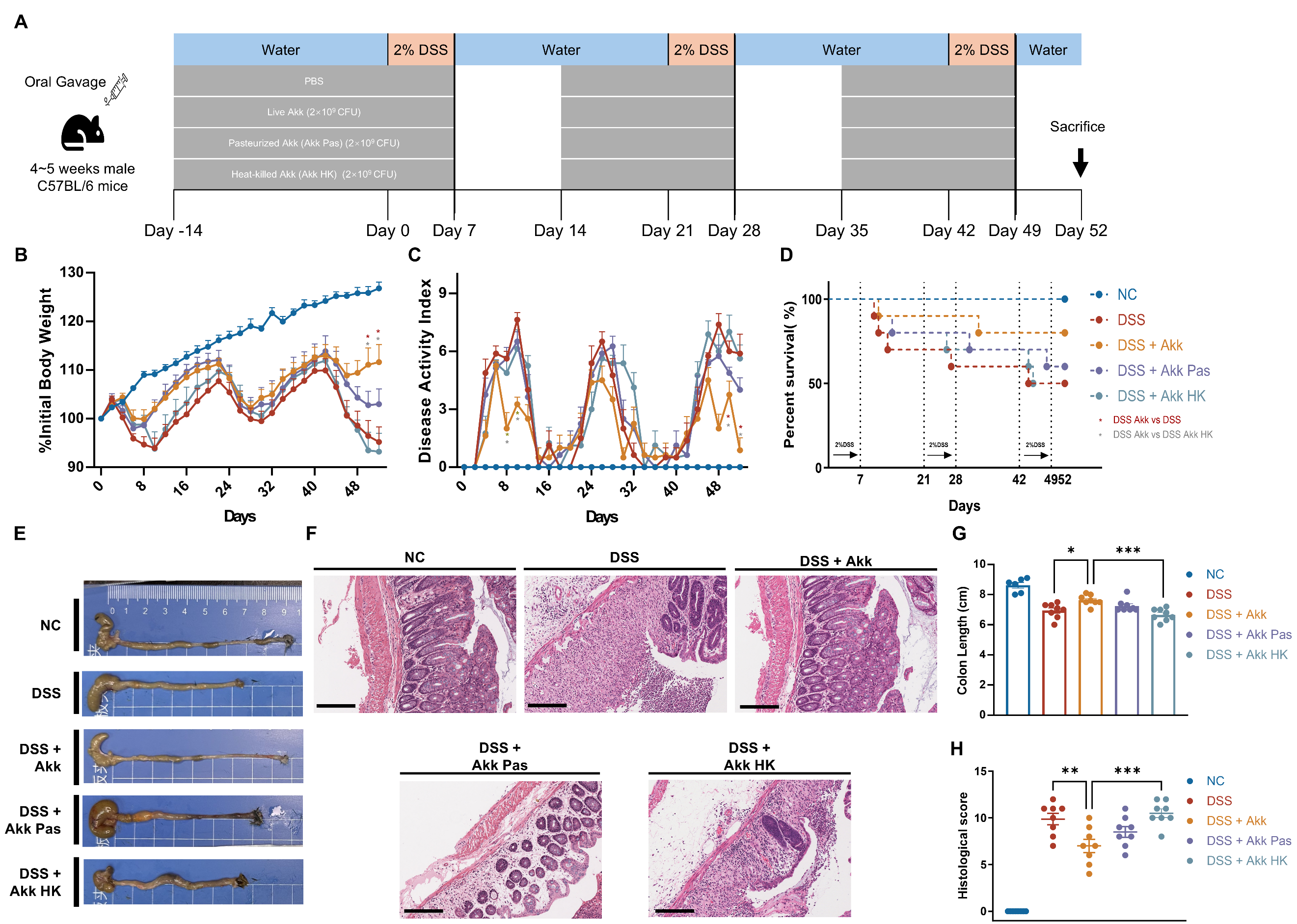


**Figure S1: Live Akk alleviates DSS-induced chronic colitis.**

(A) Mice (n = 8-10) underwent cycles of 14 days drinking water, 7 days 2% DSS water, executed thrice, suspended by a final 3-day water-only period. Mice received continuous drinking water for 52 days to serve as negative control. These mice were orally administered Akk (Live, Pasteurized, Heat-killed) or PBS 14 days prior to the first cycle and during each cycle, and 7 days before the subsequent two cycles.

(B to D) Percentage of weight loss (B), disease activity index (C), and survival curve (D) were recorded for each treatment group.

(E&F) Representative images of the colons(E) and the H&E-stained colon sections(F) from different treatment groups (scale bars: 200 μm).

(G&H) Colon length(G) and Histological score(H) of the indicated groups. * P < 0.05, ** P < 0.01, *** P < 0.001.


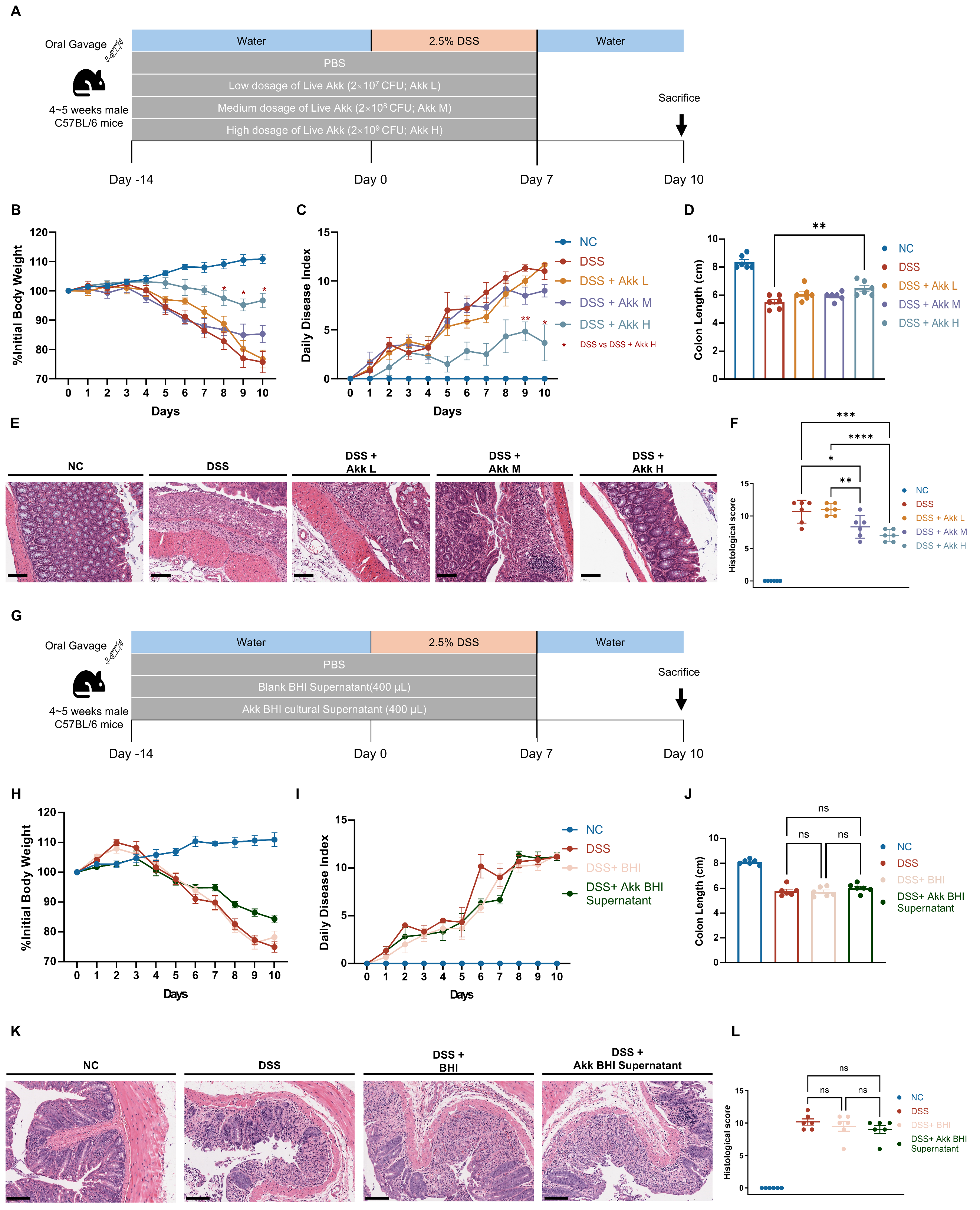


**Figure S2: High-dosage Akk supplement, but not the cultural supernatant of Akk, alleviates DSS-induced acute colitis.**

(A) Mice (n = 6) were given drinking water for 14 days, followed by 2% DSS in drinking water for 7 days, and then drinking water for 3 days. These mice were orally administered different dosages of Akk (2×10^7^, 2×10^8^, or 2×10^9^ CFU) or PBS 14 days before and during the DSS challenge.

(B to D) Percentage of weight loss (B), disease activity index (C) and colon length (D) were recorded.

(E&F) Representative H&E staining images (E) (scale bar:100 µm) and the histological score (F) for colons in mice from different treatment groups.

(G) Mice (n = 6) were given drinking water for 14 days, followed by 2% DSS in drinking water for 7 days, and then drinking water for 3 days. Mice were daily orally administered blank or AKK BHI cultural supernatants, or PBS as a control, for 14 days before and during the DSS challenge.

(H to I) Percentage of weight loss (H), disease activity index (I), and colon length (J) were recorded.

(K&L) Representative H&E staining images (K) (scale bar:100 µm) and the histological score(L) for colons in mice from the indicated groups. * P < 0.05, ** P < 0.01, *** P < 0.001, **** P < 0.0001.


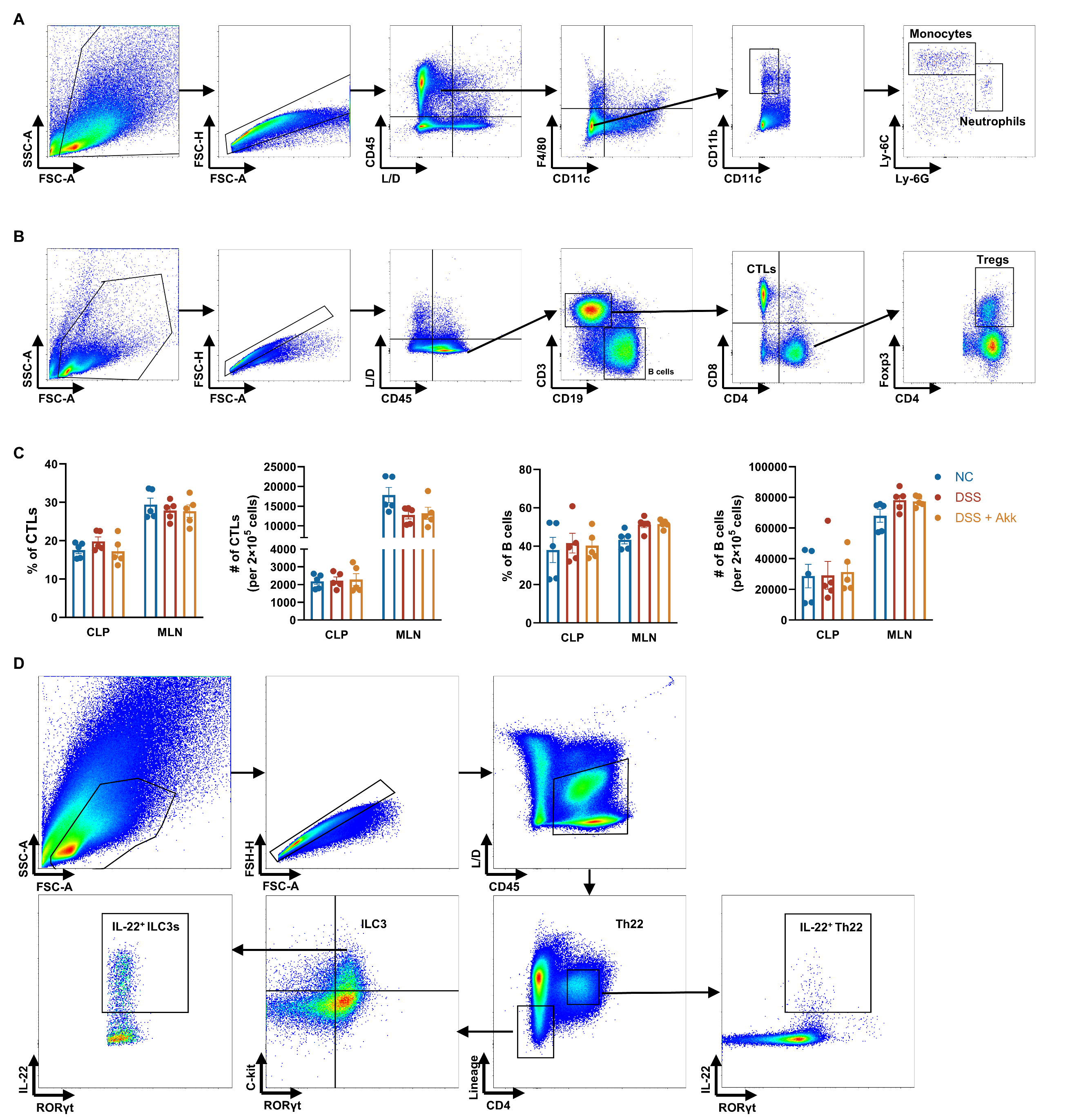


**Figure S3: related to Figure 2.**

(A&B) The gating strategy for flow cytometry analysis of CD45^+^F4/80^-^CD11c^-^CD11b^+^Ly6C^+^Ly6G^-^ monocytes (A), CD45^+^F4/80^-^CD11c^-^CD11b^+^Ly6C^-^Ly6G^+^ neutrophils (A), CD45^+^CD3^-^CD19^+^ B cells (B), CD45^+^CD3^+^CD19^-^CD4^-^CD8^+^ CTLs (B), and CD45^+^CD3^+^CD19^-^CD4^+^CD8^-^Foxp3^+^ Tregs (B).

(C) The frequencies and absolute numbers of CTLs and B cells analyzed by flow cytometry in the CLP and MLN of untreated mice and acute DSS-treated mice with gavage of PBS or Akk (n = 5).

(D) Gating strategy for flow cytometry analysis of CD45^+^Lin^-^CD4^-^C-kit^+^RORγt^+^IL-22^+^ ILC3 cells and CD45^+^Lin^+^CD4^+^RORγt^+^IL-22^+^ Th cells.

**
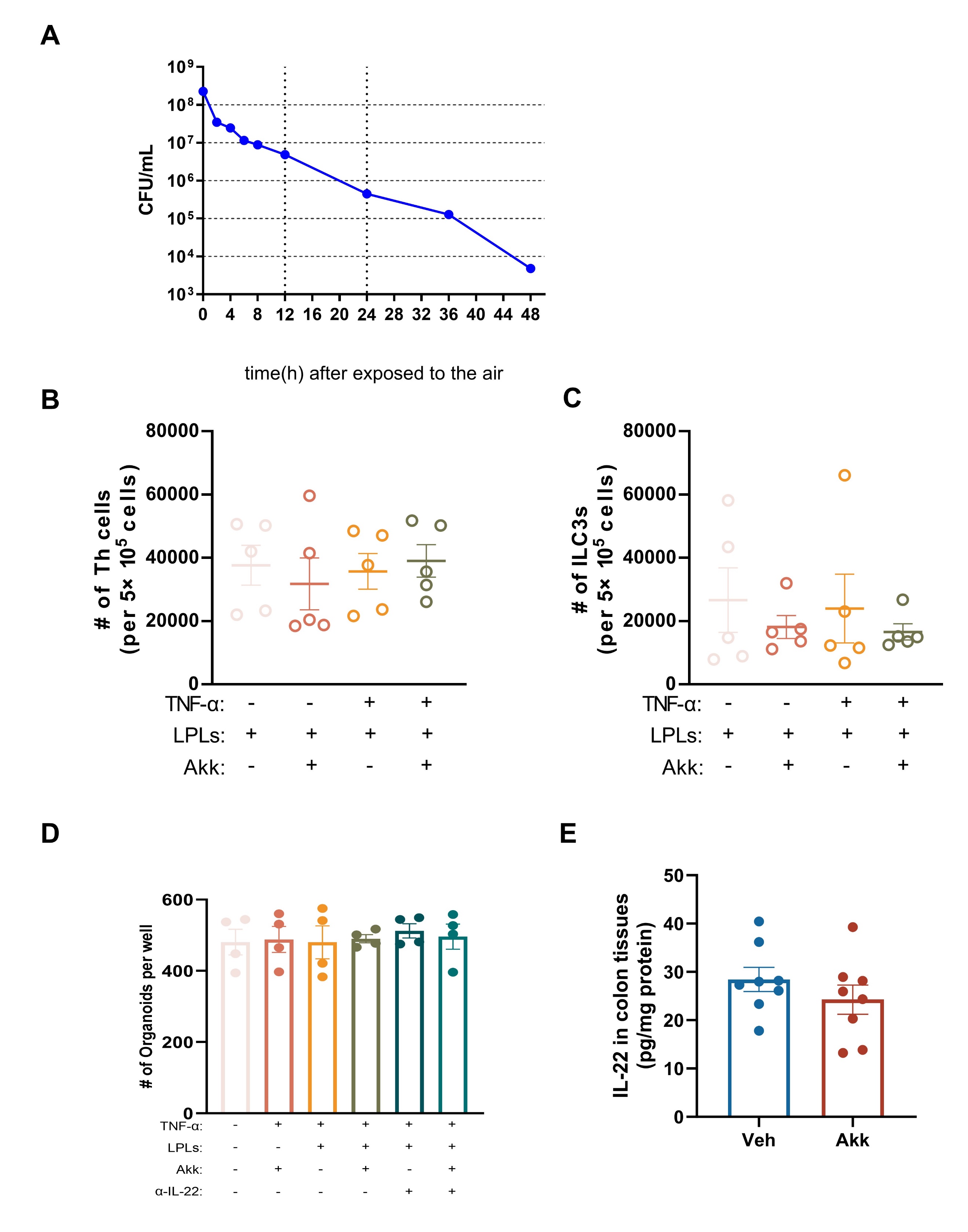
**

**Figure S4: related to Figure 3.**

(A) Akk was cultured in IMDM at 37°C with 5% CO_2_ in different time period and then incubated anaerobically on BHA plates for bacterial counting.

(B&C) The absolute numbers of Th cells (A) and ILC3 cells (B) in CLP was analyzed by flow cytometry with/without TNF-α or Akk treatment (n = 5).

(D) The absolute number of total organoids per well in different treatment groups was counted (n = 4).

(E) After 14 days of oral gavage with either Akk or Veh, mice were euthanized, colon tissues harvested, and IL-22 levels were assayed using ELISA (n = 8).


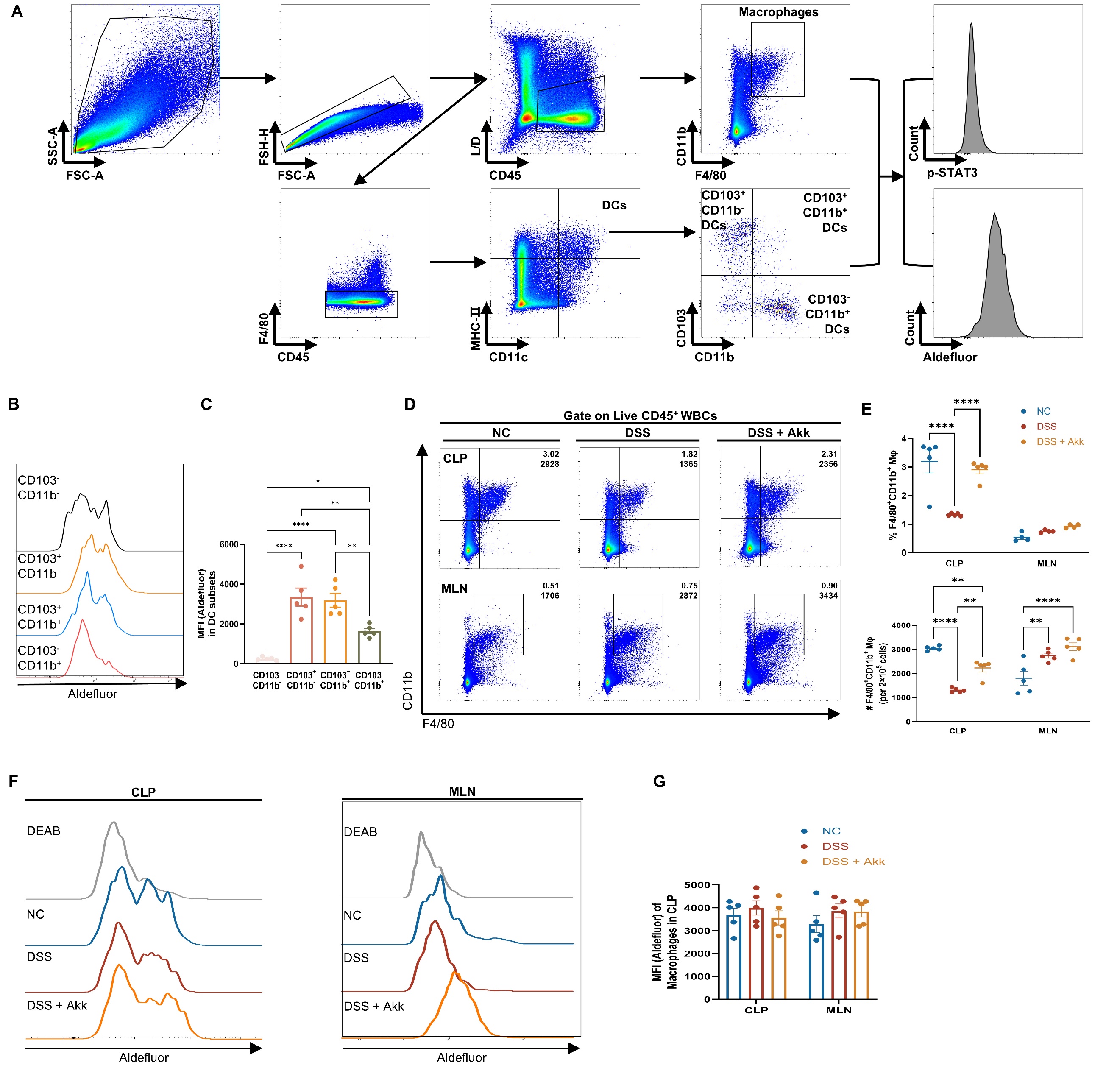


**Figure S5: related to Figure 4.**

(A) Gating strategy of CD45^+^F4/80^+^CD11c^-^CD11b^+^ macrophages, CD45^+^F4/80^-^CD11c^+^MHC-Ⅱ^+^ DCs and different subsets of CLP or MLN DCs for flow cytometry. Further analysis includes Aldefluor assay and p-STAT3 staining on total or different subsets of DCs and macrophages.

(B&C) Representative images(B) and quantitative analysis(C) for FACS Aldefluor assay of different CLP DC subsets in acute DSS-treated mice (n = 5).

(D&E) Representative flow plots of macrophages(D) in the CLP (upper) and MLN (below) from different treatment groups by Flow cytometry. Frequencies and absolute numbers of these specific populations(E) were determined (n = 5).

(F&G) Representative images(F) and quantitative analysis(G) for FACS Aldefluor assay of macrophages within the CLP and MLN (n = 5). * P < 0.05, ** P < 0.01, *** P < 0.001, **** P < 0.0001.


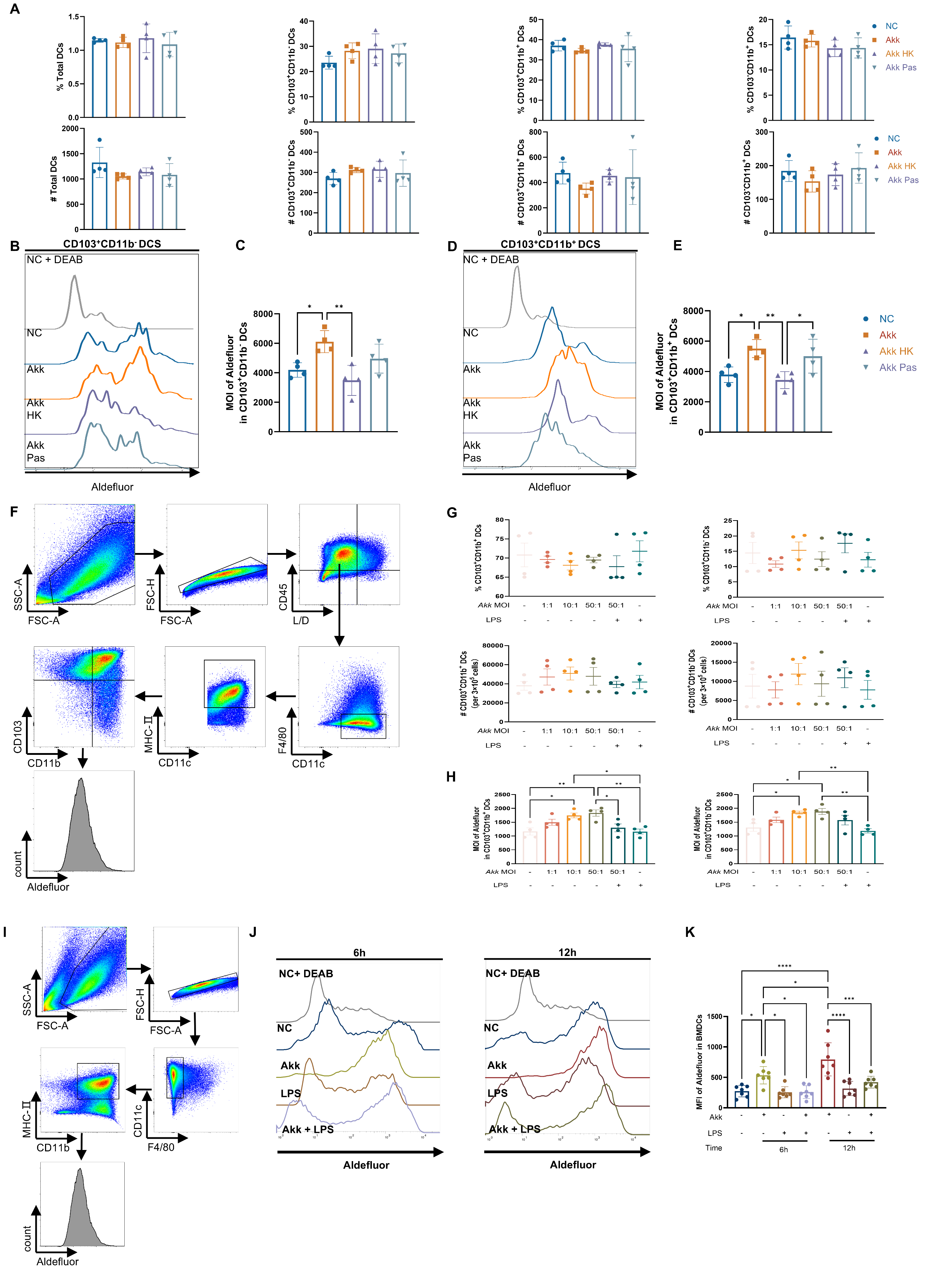


**Figure S6: Akk enhances retinoic acid synthesis in DCs *in vitro*.**

(A) Colonic LPLs were exposed to different forms of Akk (live, pasteurized, heat-killed, MOI=50). Frequencies and quantities of total or specific subsets of DCs and macrophages within these designated cells were assessed by Flow cytometry (n = 4).

(B to E) Representative images and quantitative analysis for the FACS Aldefluor assay of CD103^+^CD11b^-^ DCs (B&C) and CD103^+^CD11b^+^ DCs (D&E) in these specific cells (n = 4).

(F to H) FLT3L-derived BMDCs were stimulated with Akk (MOI 1, 10, 50) or LPS alone or in combination. Following 12 hours, flow cytometry analysis (F) was implemented, the frequencies and absolute numbers of CD103^+^CD11b^-^ DCs and CD103^+^CD11b^+^ DCs within these specified cells were determined (G). Quantitative statistical assessment of the Mean Fluorescence Intensity (MFI) of Aldefluor across different treatment groups was carried out (H) (n = 4).

(I to K) GM-CSF-derived BMDCs were exposed to Akk or LPS, either individually or in combination. After 6 or 12 hours, flow cytometry analysis was performed (I), along with representative images (J) and quantitative evaluation (K) of the MFI of Aldefluor in the indicated cells (n = 7). * P < 0.05, ** P < 0.01, *** P < 0.001, **** P < 0.0001.


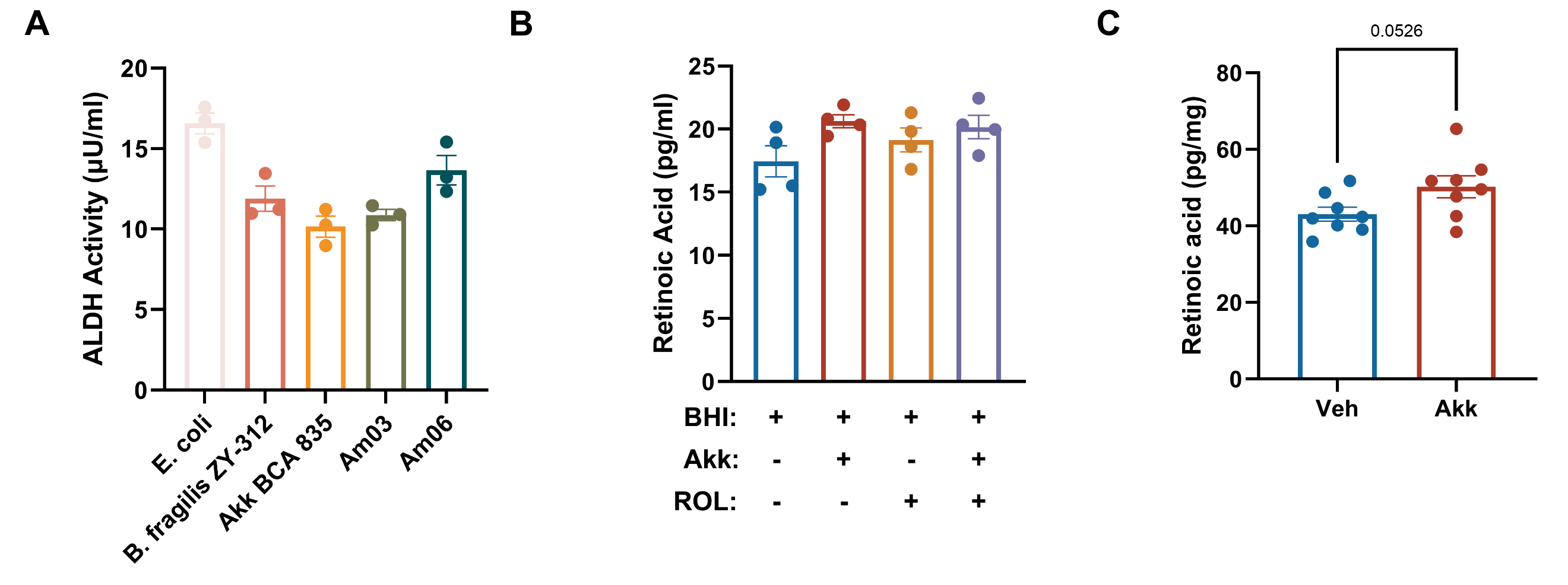


**Figure S7: Akk does not play a direct role in the synthesis of retinoic acid *in vitro*.** (A)The ALDH activity was detected in 5 different commensal species (n = 3).

(B)Quantification of retinoic acid (RA) by ELISA was performed in the blank or Akk cultural BHI supernatant treated with/without ROL (n = 4).

(E) After 14 days of oral gavage with either Akk or Veh, mice were euthanized, colon tissues harvested, and retinoic acid levels were assayed using ELISA (n = 8).


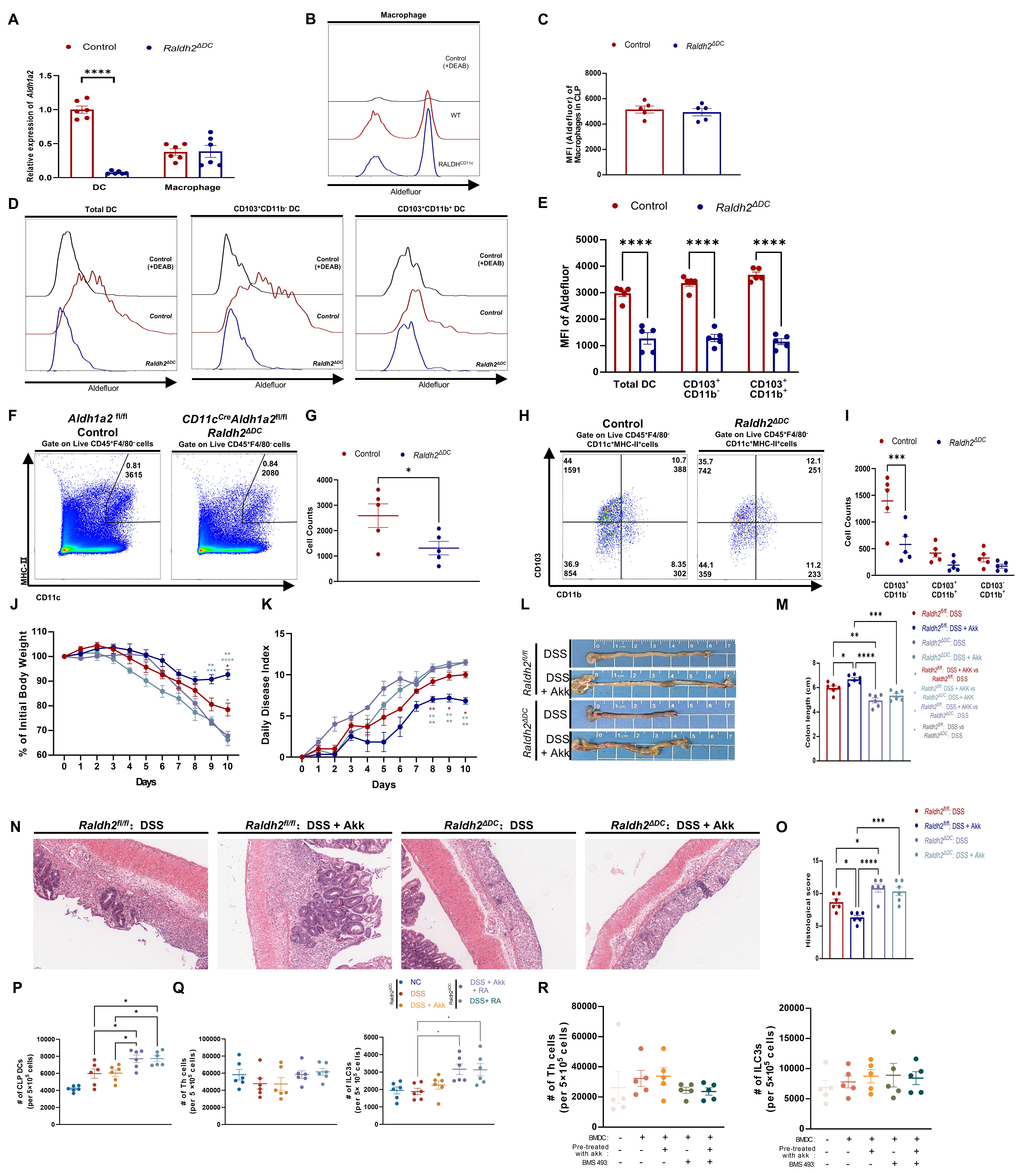


**Figure S8: Related to Figure 5.**

(A) Expression of *Aldh1a2* was evaluated in CD45^+^F4/80^-^CD11c^+^ DCs AND CD45^+^F4/80^+^CD11c^-^ macrophages from CLP in Cd11c cre *Aldh1a2^fl/fl^* (*Raldh2^ΔDC^)* and *Aldh1a2^fl/fl^* (*Raldh2^fl/fl^*, Short for Control) littermate mice.

(B&C) Representative images(B) and quantitative analysis(C) of CLP macrophages in these indicated mice by FACS Aldefluor assay (n = 5).

(D&E) Representative images(D) and quantitative analysis(E) were conducted for the FACS Aldefluor assay of total CLP DCs and CD103^+^CD11b^-^ CLP DCs and CD103^+^CD11b^+^ CLP DCs in *Raldh2^ΔDC^* and control mice (n = 5).

(F&G) Representative images(F) and quantitative analysis(G) of CLP total DCs in these indicated mice (n = 5).

(H&I) Representative images(H) and quantitative analysis(I) of different CLP DC subsets in these indicated mice (n = 5).

(J) Quantitative analysis of total CLP DCs among *Raldh2^ΔDC^* mice, which were untreated or treated by acute DSS with gavage of vehicle, Akk alone or together with RA (n = 5).

(K) Quantitative analysis of total CLP Th cells and ILC3s among these indicated mice (n = 5).

(L) Quantitative analysis of total CLP Th cells and ILC3s among cells indicated in Figure 5O to Q (n = 5).

(M to P) The pathology of colitis was evaluated among *Raldh2^fl/fl^* and *Raldh2^ΔDC^* mice, which were treated by acute DSS with gavage of vehicle or Akk, by body weight changes(M), disease activity index (N), colon lengths (O&P) (n = 6).

(Q&R) Representative H&E staining images (Q) (scale bar:100 µm) and the histological score(R) for colons in mice pretreated from different treatment groups (n = 6). * P < 0.05, ** P < 0.01, *** P < 0.001, **** P < 0.0001.


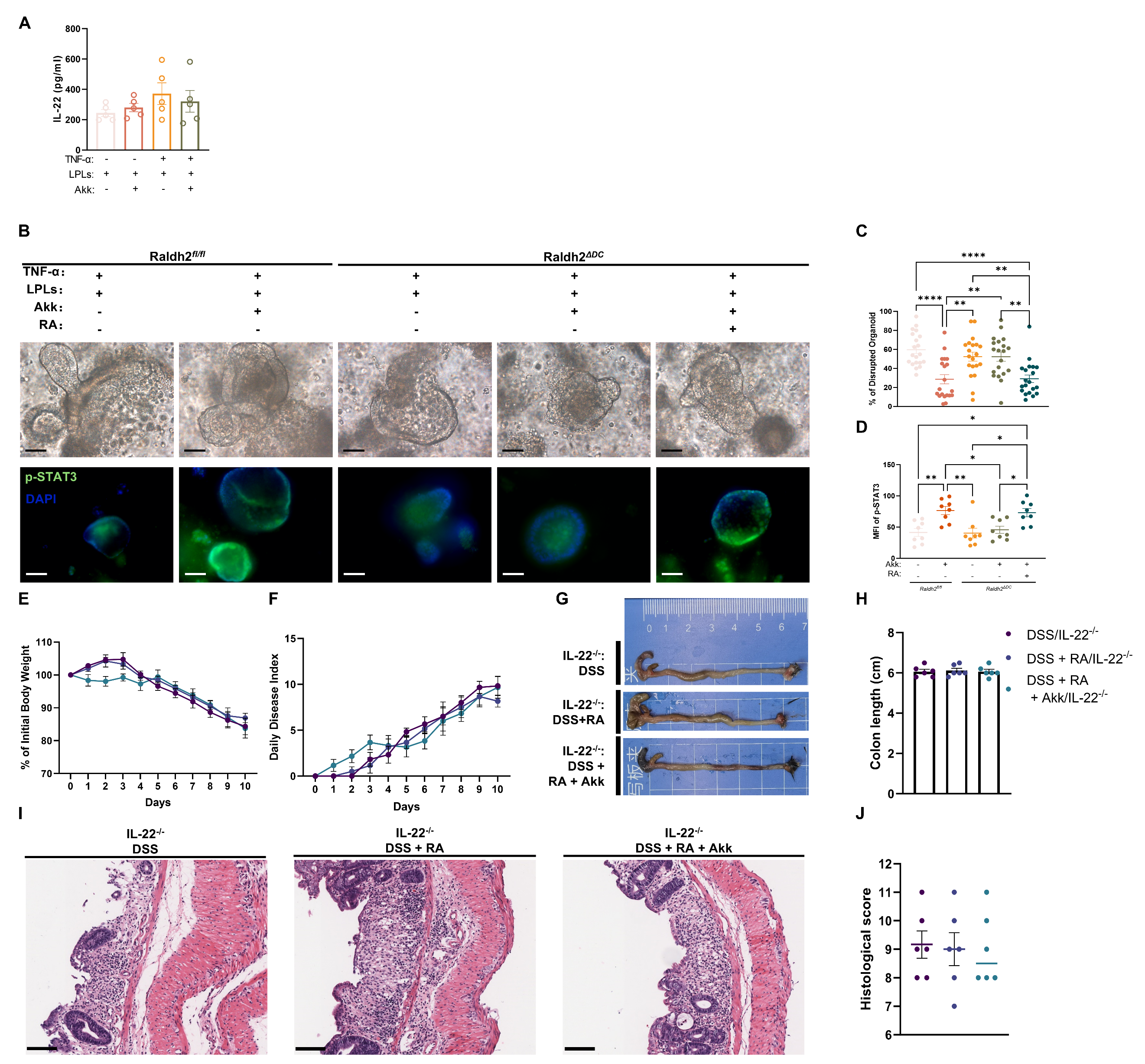


**Figure S9: Related to Figure 5.**

(A) Colonic LPLs derived from *Raldh2^ΔDC^* mice were treated with or without TNF-α or Akk (n = 5) for 12h. The IL-22 expression of cultured supernatants in these four groups was determined by ELISA.

(B to D) Colon organoids derived from *Raldh2^fl/fl^* and *Raldh2^ΔDC^* mice were cultured with or without Akk or RA in the presence of TNF-α and LPLs for 12h. The morphologies of organoids (upper) and the immunofluorescence analysis of p-STAT3 (below; p-STAT3 staining in green, DAPI staining in blue) were evaluated by light or fluorescence microscopy(B). The relative number of organoids with altered morphology (C) (n = 4, randomly select five fields of view for observation in each well) and the average fluorescence intensity of p-STAT3 (D) were quantitatively assessed (n = 8 organoids per group).

(E to J) The pathology of colitis was evaluated among IL-22^-/-^ mice, which were treated by acute DSS with gavage of vehicle, Akk alone or together with RA, by body weight changes(E), disease activity index (F), colon lengths (G&H) (n = 6).

(I&J) Representative H&E staining images (I) (scale bar:100 µm) and the histological score(J) for colons in mice pretreated from different treatment groups (n = 6). * P < 0.05, ** P < 0.01, *** P < 0.001, **** P < 0.0001.


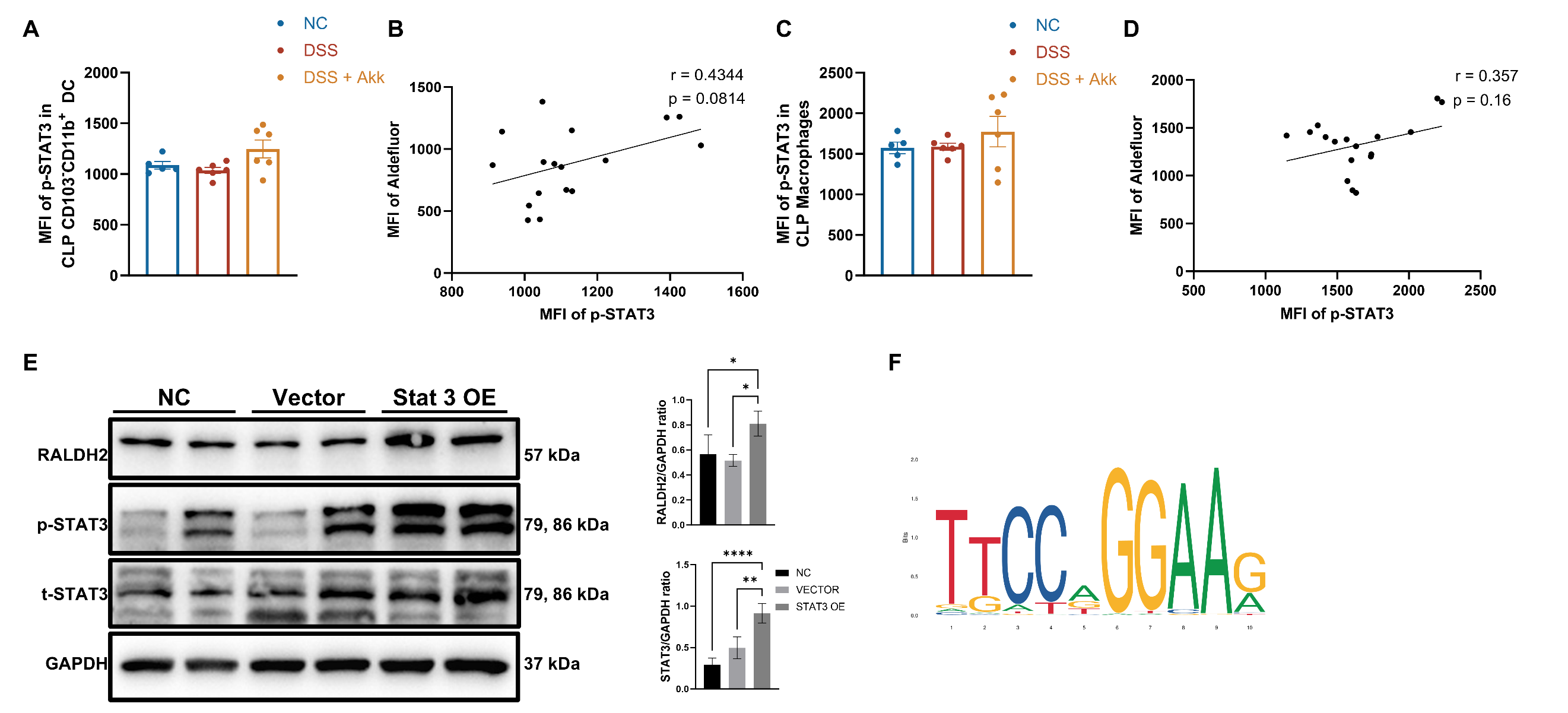


**Figure S10: Related to Figure 6.**

(A)Quantitative analysis of Flow-cytometric analysis for p-STAT3 staining in CLP CD103^-^CD11b^+^ DCs from untreated mice and acute DSS-treated mice with gavage of PBS or Akk (n = 5 - 6).

(B) A correlation analysis was conducted on CLP CD103^-^CD11b^+^ DCs to determine the relationship between the MFI of p-STAT3 and Aldefluor (n = 17).

(C)Quantitative analysis of Flow-cytometric analysis for p-STAT3 staining in CLP macrophages from untreated mice and acute DSS-treated mice with gavage of PBS or Akk (n = 5 - 6).

(D) A correlation analysis was conducted on CLP macrophages to determine the relationship between the MFI of p-STAT3 and Aldefluor (n = 17).

(E) Western blot analysis of RALDH2, p-STAT3 and STAT3 in BMDCs transfected with the vector or STAT3 overexpression (STAT3 OE) plasmids; Relative protein levels of indicated proteins were quantified to GAPDH.

(F) A conserved STAT3-binding motif was predicted by JASPAR. * P < 0.05, ** P < 0.01, *** P < 0.001, **** P < 0.0001.


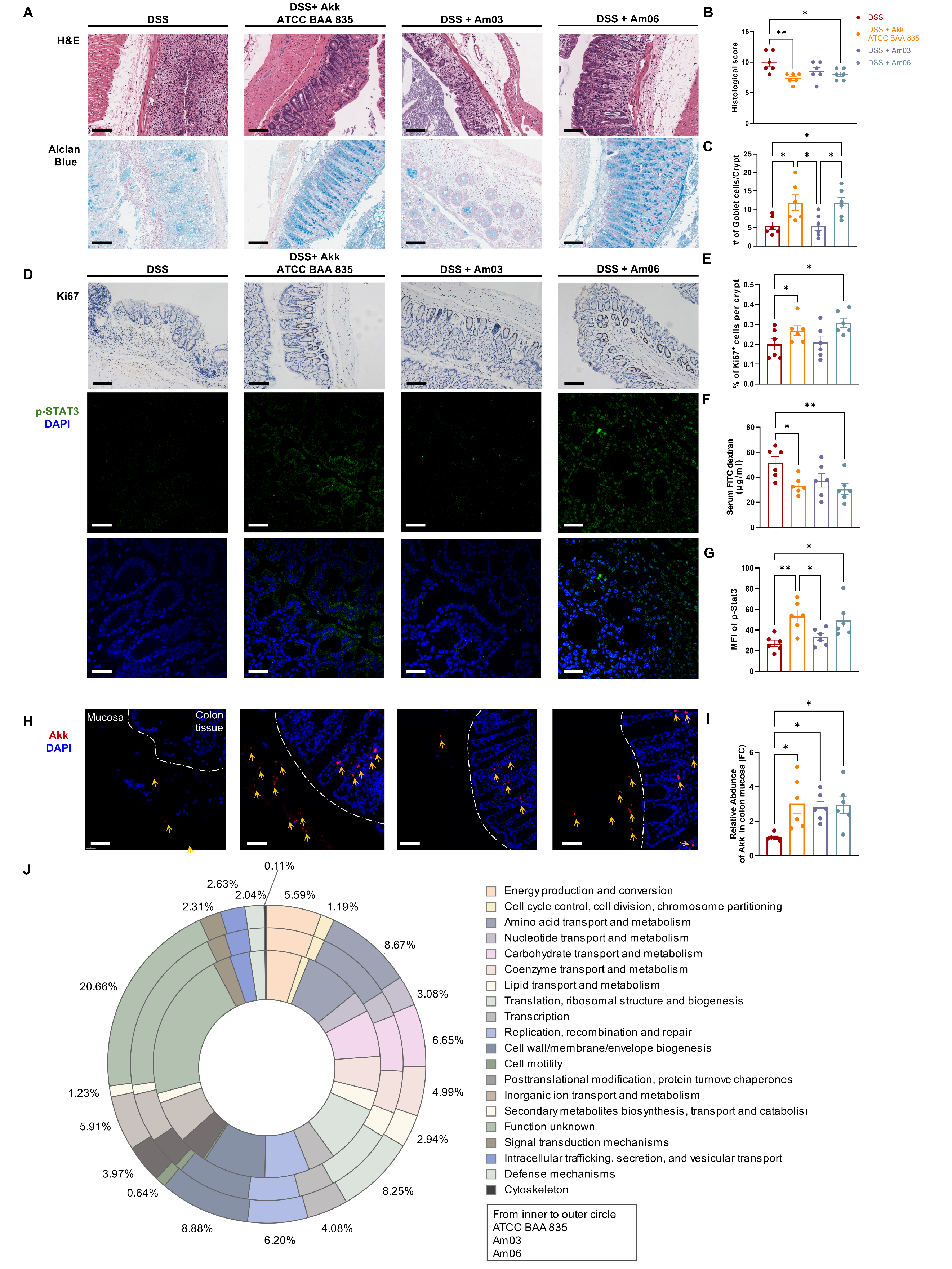


**Figure S11: Related to Figure 7.**

(A) Representative images of H&E stained(upper) and Alcian blue stained images (below) in the colon sections of 4 acute colitis groups with PBS, Akk ATCC BAA835，Am03, or Am06 gavage respectively (scale bars: 100 μm).

(B&C) Histological score (B) and quantitative analysis of the number of goblet cells in the crypt (C) (n = 6).

(D) Representative IHC images of Ki-67 immunostaining (upper) and immunofluorescence images of p-STAT3 staining in the colon sections of indicated groups (below, p-STAT3 staining in green, DAPI staining in blue; scale bars: 100 μm).

(E) Assessment of intestinal permeability by evaluating the concentration of FITC-dextran in mouse serum from different treatment groups (n = 6).

(F&G) The quantitative analysis of the number of ki67^+^ cells in the crypt (E) and the average median fluorescent intensity (MFI) of p-STAT3(F) were assessed using ImageJ (n = 6).

(H&I) Confocal Microscopy of *A. muciniphila*(H): Bacteria (red) are highlighted by a yellow arrow; DAPI staining appears in blue (Scale bar: 50 μm). (I)The relative abundance of AKK in the colonic mucosa was determined by q-PCR (n = 6).

(J) COG classification of the orthologous genes of Akk BAA 835, Am03 and Am06. For each core function, the average percentage among the 3 Akk species genomes is indicated. * P < 0.05, ** P < 0.01, *** P < 0.001.


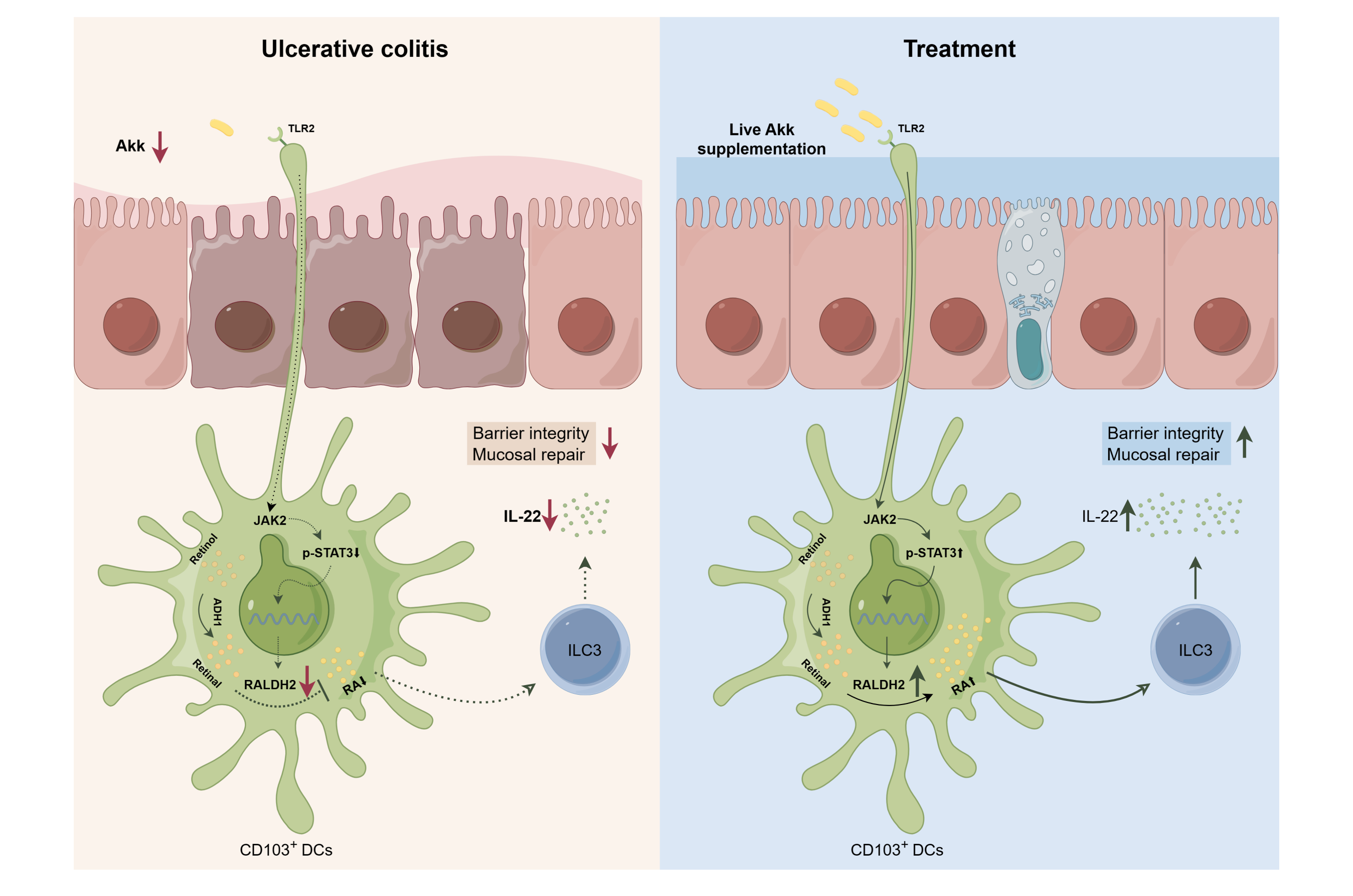


**Figure S12: Model showing how Akkermansia muciniphila（Akk） enhances dendritic cell retinoic acid synthesis to modulate IL-22 activity and mitigate colitis in mice.**

In this model, supplementation of live Akk decreased in UC promotes retinoic acid synthesis in DCs through the STAT3 pathway, leading to enhanced secretion of IL-22 by ILC3s，thereby enhancing the integrity of the colonic mucosal barrier and facilitating mucosal repair in DSS-induced colitis.

**Supplementary Tables S1-5**

**Table 1 - Top 30 upregulated and downregulated proteins (DSS + Akk vs. DSS, FDR< 0.05)**

| **Accession** | **Protein name** | **Fold change** | **P-value** | **log2FC** |
| --- | --- | --- | --- | --- |
| **Upregulation** | |  |  |  |
| Q545C3 | CDK4 | 2.949819 | 3.31E-06 | 1.560627 |
| Q9D8W7 | OCIAD2 | 2.80449 | 0.012125 | 1.487738 |
| P63163 | SNRPN | 2.765205 | 0.003443 | 1.467387 |
| Q8VC42 | 3110002H16Rik | 2.736963 | 7.09E-07 | 1.452576 |
| Q5SSW2 | PSME4 | 2.684661 | 1.55E-05 | 1.42474 |
| O08709 | PRDX6 | 2.653093 | 2E-06 | 1.407675 |
| Q9CWL8 | CTNNBL1 | 2.63696 | 8.13E-05 | 1.398875 |
| P55095 | GCG | 2.411246 | 0.003572 | 1.269779 |
| Q921G8 | TUBGCP2 | 2.359506 | 1.75E-06 | 1.238485 |
| Q62165 | DAG1 | 2.232878 | 4.61E-07 | 1.158904 |
| Q62273 | SLC26A2 | 2.190807 | 8.46E-05 | 1.131462 |
| Q9CXY6 | ILF2 | 2.178791 | 7.42E-09 | 1.123528 |
| I7HFT9 | hist1h1t | 2.174421 | 0.001638 | 1.120631 |
| Q5EBG8 | AU022252 | 2.159512 | 0.003438 | 1.110705 |
| Q62148 | ALDH1A2 | 2.110061 | 8.49E-06 | 1.077285 |
| Q80TL7 | MON2 | 2.080029 | 1.62E-06 | 1.056604 |
| P30275 | Ckmt1 | 2.054145 | 0.000537 | 1.038538 |
| P97499 | TEP1 | 2.049563 | 3.74E-06 | 1.035316 |
| A2AIY3 | INSL5 | 2.039097 | 2.79E-06 | 1.027931 |
| A0A0G2JG52 | AIM1 | 2.021132 | 1.09E-06 | 1.015163 |
| Q3UYV9 | NCBP1 | 1.987491 | 5.88E-06 | 0.990948 |
| Q8BIW1 | PRUNE1 | 1.974835 | 6.19E-07 | 0.981732 |
| Q8BP67 | RPL24 | 1.947336 | 0.00025 | 0.961502 |
| Q9CR16 | PPID | 1.93699 | 0.007864 | 0.953817 |
| A0A075B5T3 | Ighv6-6 | 1.935742 | 0.01564 | 0.952886 |
| Q80XN0 | BDH1 | 1.923459 | 0.007132 | 0.943703 |
| Q8K419 | LGALS4 | 1.921954 | 0.007771 | 0.942574 |
| Q68FL4 | AHCYL2 | 1.919569 | 0.003763 | 0.940782 |
| P51885 | LUM | 1.895838 | 8.51E-05 | 0.922836 |
| Q3UKA4 | ADH1 | 1.875464 | 7.63E-06 | 0.907247 |
| **Downregulation** |  |  |  |  |
| P07091 | S100A4 | 0.633448 | 0.045521 | -0.6587 |
| Q9QYJ3 | DNAJB1 | 0.628532 | 0.000143 | -0.66994 |
| P52633 | STAT6 | 0.624472 | 3.84E-08 | -0.67929 |
| F6T2Z7 | CALD1 | 0.623386 | 0.00011 | -0.6818 |
| B1AWT3 | RRAGD | 0.616897 | 9.21E-08 | -0.6969 |
| Q9CQ01 | Rnaset2b | 0.616576 | 2.11E-07 | -0.69765 |
| Q8BH69 | SEPHS1 | 0.614711 | 3.24E-06 | -0.70202 |
| Q80YQ1 | THBS1 | 0.611816 | 0.001958 | -0.70883 |
| O08692 | Ngp | 0.608883 | 0.023357 | -0.71576 |
| Q9JI39 | abcb10 | 0.608153 | 7.08E-06 | -0.71749 |
| Q545V3 | ENO2 | 0.606038 | 3.82E-06 | -0.72252 |
| A0A0G2JFT8 | RUFY3 | 0.605092 | 4.38E-07 | -0.72477 |
| P97346 | NXN | 0.596863 | 1.57E-06 | -0.74453 |
| O08638 | MYH11 | 0.574688 | 0.000188 | -0.79915 |
| Q4JFI8 | APCS | 0.573058 | 0.001351 | -0.80325 |
| Q91YL7 | CWH43 | 0.565275 | 7.88E-05 | -0.82297 |
| P50172 | HSD11B1 | 0.560642 | 7.93E-05 | -0.83485 |
| P10711 | TCEA1 | 0.555032 | 2.63E-10 | -0.84936 |
| I3RSH5 | WWP2 | 0.546685 | 2.8E-07 | -0.87122 |
| Q9JM95 | ST6GALNAC6 | 0.543896 | 9.82E-06 | -0.8786 |
| D3YUE2 | PCOLCE | 0.514361 | 0.000115 | -0.95915 |
| P18419 | Svs4 | 0.505469 | 0.005412 | -0.98431 |
| Q9WV54 | ASAH1 | 0.493238 | 7.85E-16 | -1.01964 |
| J3QPG5 | PSAP | 0.485898 | 3.75E-08 | -1.04127 |
| B1AT82 | PRPSAP1 | 0.474636 | 8.32E-08 | -1.07511 |
| Q8BZA9 | TIGAR | 0.473395 | 0.000105 | -1.07888 |
| A0A0R4J071 | TGM4 | 0.424997 | 3.04E-05 | -1.23448 |
| F2Z472 | Svs3a | 0.38733 | 1.82E-06 | -1.36836 |
| Q8BS30 | Svs2 | 0.300724 | 6.56E-10 | -1.73349 |
| Q6WIZ7 | Svs1 | 0.225582 | 5.25E-07 | -2.14828 |

**Table S2. The genomic features of Akk ATCC BAA 835, Am03 and Am06**

| **Feature** | **ATCC BAA 835** | **Am03** | **Am06** |
| --- | --- | --- | --- |
| Genome size (bp) | 2,664,100 | 2,825,705 | 2,640,251 |
| G+C content | 55.74% | 55.31% | 55.73% |
| Number of 5S rRNA genes | 3 | 3 | 3 |
| Number of 16S rRNA genes | 3 | 3 | 3 |
| Number of 23S rRNA genes | 3 | 3 | 3 |
| Number of Coding Sequences | 2182 | 2,193 | 2,144 |
| Protein-coding genes with assigned EggNOG | 1779  (81.53%) | 1757  (80.12%) | 1774  (82.74%) |

**Table S3. The strain-specific Coding Sequences(CDS) between Akk ATCC BAA 835, Am03 and Am06 strains(Identity > 60%)**

| **Locus tag** | **eggNOG OGs** | **Description** |
| --- | --- | --- |
| **ATCC BAA 835&Am06** | | |
| *Amuc_1355* | COG5525 | Phage terminase large subunit (GpA) |
| *Amuc_1335* | COG5511 | Phage portal protein, lambda family |
| *Amuc_0600* | COG4974 | xerD1, Belongs to the 'phage' integrase family |
| *Amuc_1115* | COG4974 | xerD1, Belongs to the 'phage' integrase family |
| *Amuc_1378* | COG4974 | Belongs to the 'phage' integrase family |
| *Amuc_0602* | COG4642 | regulation of ryanodine-sensitive calcium-release channel activity |
| *Amuc_1114* | COG4625 | pathogenesis |
| *Amuc_0623* | COG4409 | exo-alpha-(2->6)-sialidase activity |
| *Amuc_0146* | COG3669 | Alpha-L-fucosidase |
| *Amuc_1367* | COG3617 | BRO family, N-terminal domain |
| *Amuc_1522* | COG3587 | res, Type III restriction enzyme, res subunit |
| *Amuc_2010* | COG3513 | cas9, defense response to virus |
| *Amuc_2008* | COG3512 | cas2, CRISPR |
| *Amuc_0697* | COG3507 | Belongs to the glycosyl hydrolase 43 family |
| *Amuc_2164* | COG3325 | Glycosyl hydrolases family 18 |
| *Amuc_2086* | COG3307 | -O-antigen |
| *Amuc_1143* | COG3209 | PFAM YD repeat-containing protein |
| *Amuc_1145* | COG3209 | PFAM YD repeat-containing protein |
| *Amuc_2154* | COG3209 | self proteolysis |
| *Amuc_0605* | COG2951 | lytic endotransglycosylase activity |
| *Amuc_1354* | COG2826 | transposase and inactivated derivatives, IS30 family |
| *Amuc_1544* | COG2755 | PFAM lipolytic protein G-D-S-L family |
| *Amuc_2096* | COG2244 | polysaccharide biosynthetic process |
| *Amuc_1521* | COG2189 | PFAM DNA methylase N-4 N-6 domain protein |
| *Amuc_1808* | COG2159 | Amidohydrolase |
| *Amuc_0329* | COG1846 | Putative ATP-dependent DNA helicase recG C-terminal |
| *Amuc_0643* | COG1807 | 4-amino-4-deoxy-L-arabinose transferase activity |
| *Amuc_1690* | COG1669 | Nucleotidyltransferase substrate binding protein like |
| *Amuc_1691* | COG1669 | nucleotidyltransferase activity |
| *Amuc_1123* | COG1657 | Prenyltransferase and squalene oxidase repeat |
| *Amuc_2056* | COG1633 | Catalyzes the formation of the isocyclic ring in chlorophyll biosynthesis. |
| *Amuc_0624* | COG1609 | Periplasmic binding protein-like domain |
| *Amuc_1361* | COG1598 | sequence-specific DNA binding |
| *Amuc_2009* | COG1518 | cas1, maintenance of DNA repeat elements |
| *Amuc_0628* | COG1403 | endonuclease activity |
| *Amuc_0328* | COG1196 | nuclear chromosome segregation |
| *Amuc_1348* | COG1196 | nuclear chromosome segregation |
| *Amuc_2095* | COG1143 | NDH-1 shuttles electrons from NADH, via FMN and iron- sulfur (Fe-S) centers, to quinones in the respiratory chain. |
| *Amuc_1676* | COG1002 | Type III restriction enzyme res subunit |
| *Amuc_0325* | COG0863 | N-4 methylation of cytosine |
| *Amuc_2097* | COG0778 | Nitroreductase family |
| *Amuc_1914* | COG0732 | type I restriction modification DNA specificity domain |
| *Amuc_1005* | COG0667 | Aldo/keto reductase family |
| *Amuc_1006* | COG0667 | Aldo/keto reductase family |
| *Amuc_1807* | COG0667 | tas, Aldo Keto reductase |
| *Amuc_1336* | COG0616 | Peptidase family S49 |
| *Amuc_1913* | COG0610 | Type I restriction enzyme R protein N terminus (HSDR_N) |
| *Amuc_0330* | COG0582 | DNA integration |
| *Amuc_1519* | COG0553 | SNF2 family N-terminal domain |
| *Amuc_2093* | COG0463 | PFAM glycosyl transferase family 2 |
| *Amuc_2094* | COG0463 | Glycosyl transferase, family 2 |
| *Amuc_1735* | COG0454 | -acetyltransferase |
| *Amuc_2088* | COG0438 | Glycosyl transferases group 1 |
| *Amuc_2089* | COG0438 | Glycosyl transferase 4-like domain |
| *Amuc_2082* | COG0438 | lsgC, transferase activity, transferring glycosyl groups |
| *Amuc_2083* | COG0438 | lsgC, transferase activity, transferring glycosyl groups |
| *Amuc_2084* | COG0438 | gtf1, transferase activity, transferring glycosyl groups |
| *Amuc_2090* | COG0438 | lsgC, transferase activity, transferring glycosyl groups |
| *Amuc_1377* | COG0305 | DnaB-like helicase C terminal domain |
| *Amuc_1915* | COG0286 | N-6 DNA Methylase |
| *Amuc_1748* | COG0240 | odh, glycerol-3-phosphate dehydrogenase [NAD(P)+] activity |
| *Amuc_1097* | COG0237 | coaE, Catalyzes the phosphorylation of the 3'-hydroxyl group of dephosphocoenzyme A to form coenzyme A |
| *Amuc_2092* | COG0110 | maltose O-acetyltransferase activity |
| *Amuc_2087* | 2E5B4 | Glycosyl transferase family 11 |
| *Amuc_1520* | 2DM2D | Domain of unknown function (DUF4391) |
| *Amuc_0062* | 2CJNI | ecoRIR, Type II restriction endonuclease, EcoRI family protein |
| *Amuc_1564* | 28IMK | dpnC, Dam-replacing family |
| **ATCC BAA 835&Am03** | | |
| *Amuc_1039* | COG4625 | pathogenesis |
| *Amuc_1723* | COG2911 | pathogenesis |
| *Amuc_1248* | COG0451 | fcl, Catalyzes the two-step NADP-dependent conversion of GDP- 4-dehydro-6-deoxy-D-mannose to GDP-fucose, involving an epimerase and a reductase reaction |
| **Am03&Am06** | |  |
| *Bacsa_1151* | COG3378 | Psort location Cytoplasmic |
| *AUAE01000022_gene1461* | COG1075 | Alpha/beta hydrolase family |
| **ATCC BAA 835** | |  |
| *Amuc_1722* | COG4625 | pathogenesis |
| *Amuc_1858* | COG3410 | Uncharacterized conserved protein (DUF2075) |
| *Amuc_0323* | COG3311 | DNA excision |
| *Amuc_1283* | COG3210 | Passenger-associated-transport-repeat |
| *Amuc_0983* | COG3209 | PFAM YD repeat-containing protein |
| *Amuc_2152* | COG3209 | PFAM YD repeat-containing protein |
| *Amuc_0584* | COG2911 | pathogenesis |
| *Amuc_1362* | COG1724 | mRNA binding |
| *Amuc_0741* | COG1432 | NYN domain |
| *Amuc_0308* | COG0048 | Ribosomal protein S12/S23 |
| **Am06** |  |  |
| *CFT03427_0821* | COG3392 | DNA methyltransferase |
| *BACOVA_03771* | COG3153 | Acetyltransferase (GNAT) domain |
| *KE386491_gene3360* | COG0119 | leuA, Belongs to the alpha-IPM synthase homocitrate synthase family |
| *KE386495_gene1587* | COG0119 | leuA, Catalyzes the condensation of the acetyl group of acetyl-CoA with 3-methyl-2-oxobutanoate (2-oxoisovalerate) to form 3-carboxy-3-hydroxy-4-methylpentanoate (2-isopropylmalate) |
| **Am03** |  |  |
| *CLOSPI_00232* | COG4832 | Psort location Cytoplasmic, score |
| *JPJG01000064_gene1790* | COG4096 | T5orf172 |
| *Bacsa_1150* | COG3943 | COG3943, virulence protein |
| *BACCOPRO_02597* | COG3669 | Alpha-L-fucosidase |
| *GGE_1752* | COG3649 | CRISPR-associated protein |
| *RBI_I02000* | COG3593 | Protein of unknown function (DUF2813) |
| *C900_00846* | COG3550 | HipA N-terminal domain |
| *HMPREF0240_01205* | COG3153 | Acetyltransferase (GNAT) family |
| *BT1A1_0316* | COG2189 | mod, DNA methylase |
| *JNJN01000017_gene2286* | COG1636 | queH, Catalyzes the conversion of epoxyqueuosine (oQ) to queuosine (Q), which is a hypermodified base found in the wobble positions of tRNA(Asp), tRNA(Asn), tRNA(His) and tRNA(Tyr) |
| *N177_4108* | COG1518 | cas1, CRISPR associated protein Cas1 |
| *HQ41_00970* | COG1343 | cas2, CRISPR |
| *HMPREF1545_04312* | COG1246 | Acetyltransferase (GNAT) domain |
| *Bacsa_1154* | COG1196 | plasmid recombination enzyme |
| *HG726023_gene3226* | COG1131 | AAA domain |
| *ALO_09139* | COG0841 | Belongs to the resistance-nodulation-cell division (RND) (TC 2.A.6) family |
| *NLA_14040* | COG0732 | Type I restriction modification DNA specificity domain |
| *Swol_0934* | COG0685 | metF, 5,10-methylenetetrahydrofolate reductase |
| *HMPREF9441_03722* | COG0667 | aldo keto reductase |
| *CBLK010000064_gene855* | COG0620 | metE, Catalyzes the transfer of a methyl group from 5- methyltetrahydrofolate to homocysteine resulting in methionine formation |
| *Bache_2558* | COG0610 | hsdR, Subunit R is required for both nuclease and ATPase activities, but not for modification |
| *Swol_0935* | COG0583 | Psort location Cytoplasmic |
| *Bacsa_1149* | COG0582 | COG4974 Site-specific recombinase XerD |
| *P343_12775* | COG0500 | DNA (cytosine-5-)-methyltransferase activity |
| *Bacsa_1153* | COG0358 | Toprim-like |
| *JCM15093_1705* | COG0338 | DNA N-6-adenine-methyltransferase (Dam) |
| *Bache_2554* | COG0286 | hsdM, COG0286 Type I restriction-modification system methyltransferase subunit |
| *HMPREF1008_00291* | COG0286 | restriction endonuclease |
| *HMPREF9442_00826* | COG0210 | DNA helicase |
| *BAKL01000131_gene5590* | COG0119 | leuA, Catalyzes the condensation of the acetyl group of acetyl-CoA with 3-methyl-2-oxobutanoate (2-oxoisovalerate) to form 3-carboxy-3-hydroxy-4-methylpentanoate (2-isopropylmalate) |
| *NSB1T_02735* | COG0110 | acetyltransferase, isoleucine patch superfamily |
| *BFAG_04833* | COG0030 | Belongs to the class I-like SAM-binding methyltransferase superfamily. rRNA adenine N(6)-methyltransferase family |
| *PGN_0928* | arCOG14100 | SIR2-like domain |
| *HMPREF2087_01727* | 2E7TI | TRL-like protein family |

**Table S4. The antibodies and reagents used in this study.**

| **REAGENTS or ANTIBODIES** | | **SOURCE** | **IDENTIFIER** |
| --- | --- | --- | --- |
| Anti-pSTAT3 (Tyr705) | Cell Signaling Technology | | CST#9145 |
| Anti-STAT3 (Tyr705) | Cell Signaling Technology | | CST#4904 |
| Anti-RALDH2 | NOVUS | | NBP2-92915 |
| Anti-TLR2 | Protein Tech | | 66645-1-Ig |
| Anti-pJAK2 | Cell Signaling Technology | | CST#3771 |
| Anti-JAK2 | Cell Signaling Technology | | CST#3230 |
| Anti-SOCS2 | Cell Signaling Technology | | CST#2779 |
| Anti-pmTOR | Cell Signaling Technology | | CST#2972 |
| Anti-mTOR | Cell Signaling Technology | | CST#5536 |
| Anti-GAPDH | Cell Signaling Technology | | CST#5174 |
| KI-67 | Abcam | | Ab16667 |
| Alcian blue | Biossci | | BP040 |
| Goat Anti-Rabbit IgG Antibody [H+L], FITC Conjugated | Beyotime | | A0556 |
| DAPI | Beyotime | | C1005 |
|  |  | |  |
| IL-22 Mouse Uncoated ELISA Kit | Thermo Fisher Scientific | | 88-7422-86 |
| IL-6 Mouse Uncoated ELISA Kit | Thermo Fisher Scientific | | 88-7064-88 |
| TNF alpha Mouse Uncoated ELISA Kit | Thermo Fisher Scientific | | 88-7324-88 |
| IL-17 Mouse Uncoated ELISA Kit | Thermo Fisher Scientific | | 88-7324-88 |
| IL-10 Mouse Uncoated ELISA Kit | Thermo Fisher Scientific | | 88-7064-88 |
| Mouse IFN gamma Uncoated ELISA Kit | Thermo Fisher Scientific | | 88-7314-88 |
| Mouse IL-12 p70 Uncoated ELISA Kit | Thermo Fisher Scientific | | 88-7121-22 |
| IL-23 Mouse Uncoated ELISA Kit | Thermo Fisher Scientific | | 88-7230-22 |
| Mouse Retinoic Acid ELISA kit | Cusabio | | CSB-EQ028019MO |
| DSS | MP Biomedicals | | 9011-18-1 |
| FITC-dextran | Sigma-Aldrich | | 46944 |
| Urine fecal occult blood test kit | Jiancheng Bioengineering Institute | | C027-1-1 |
| Retinoic acid | Selleck | | S1653 |
| Mouse IL-22 Affinity Purified Polyclonal Ab (25 ug) | R＆D system | | AF582-SP |
| Murine IL-22 | Peprotech | | 210-22-10 |
| TNF-α | Peprotech | | 315-01A-20 |
| LPS | Beyotime | | ST1470 |
| Recombinant murine Flt3 ligand | Peprotech | | 250-31L |
| GM-CSF | Peprotech | | 315-03 |
| BMS 493 | Stem cell | | 73972 |
| Stattic | Selleck | | S7024 |
| ALDH Activity Assay Kit | Abcam | | ab155893 |
| Anti-CD16/32 FcR blocker | Biolegend | | 101302 |
| Purified hamster anti-mouse CD3e | BD Biosciences | | 553058 |
| Purified hamster anti-mouse CD28 | BD Biosciences | | 553295 |
| eBioscience™ Foxp3 / Transcription Factor Staining Buffer Set | Invitrogen | | 00-5523-00 |
| eBioscience™ Protein Transport Inhibitor Cocktail (500X) | Thermo Fisher Scientific | | 00-4980-03 |
| RBC lysis buffer | Solarbio | | R1010 |
| Alexa Fluor® 700 Rat Anti-Mouse CD45 | BD Biosciences | | 560510 |
| Fixable Viability Stain 780 | BD Biosciences | | 565388 |
| Horizon™ BV605 Rat Anti-Mouse CD4 | BD Biosciences | | 563151 |
| Pharmingen™ PerCP-Cy™5.5 Mouse Lineage Antibody Cocktail,with Isotype Control | BD Biosciences | | 561317 |
| FITC anti-mouse CD117 (c-Kit) Antibody | Biolegend | | 105805 |
| Horizon™ BV421 Mouse Anti-Mouse RORγt | BD Biosciences | | 562894 |
| PE anti-mouse IL-22 Antibody | Biolegend | | 516404 |
| APC Rat Anti-Mouse F4/80 | BD Biosciences | | 566787 |
| PerCP-Cy™5.5 Rat Anti-CD11b | BD Biosciences | | 561114 |
| Horizon™ BV421 Hamster Anti-Mouse CD11c | BD Biosciences | | 562782 |
| Horizon™ BV605 Rat Anti-Mouse IA/IE | BD Biosciences | | 563413 |
| Horizon™ PE-CF594 Rat Anti-Mouse CD103 | BD Biosciences | | 565849 |
| PE anti-STAT3 Phospho (Tyr705) Antibody | Biolegend | | 651004 |
| ALDEFLUOR™ Kit for ALDH Assays | Stem cell | | 01700 |
| BV510 Rat Anti-CD11b | BD Biosciences | | 562950 |
| PE-Cy™7 Hamster Anti-Mouse CD11c | BD Biosciences | | 558079 |
| BB700 Hamster Anti-Mouse CD11C | BD Biosciences | | 566505 |
| PE-CF594 Rat Anti-Mouse F4/80 | BD Biosciences | | 565613 |
| BV605 Rat Anti-Mouse Ly-6C | BD Biosciences | | 563011 |
| PE-Cy™7 Rat Anti-Mouse Ly-6G | BD Biosciences | | 560601 |
| PerCP-Cy™5.5 Hamster Anti-Mouse CD3e | BD Biosciences | | 551163 |
| PE Rat Anti-Mouse CD8a | BD Biosciences | | 553032 |
| BV510 Rat Anti-Mouse CD19 | BD Biosciences | | 562956 |
| FOXP3 Monoclonal Antibody (FJK-16s), FITC | eBioscience | | 11-5773-82 |
| Gentle Cell Dissociation Reagent | Stem cell | | 07174 |
| IntestiCult OGM Mouse Kit | Stem cell | | 06005 |
| Matrigel® Growth Factor Reduced (GFR) Basement Membrane Matrix, Phenol red-free, LDEV-free | Corning | | 356231 |
| DMEM / F12 MEDIA with 15 mM HEPES, | Stem cell | | 36254 |
| Advanced DNA RNA Transfection Reagent | Zeta life | | AD600150 |
| Dual Luciferase Reporter Gene Assay Kit Ⅱ | Beyotime | | RG029S |
| Dispase | Roche | | 10104159001 |
| Collagenase Type IV | Vetec | | V900893 |
| Dispase Ⅱ | YEASEN | | 40104ES80 |
| SimpleChIP® Enzymatic Chromatin IP Kit (Magnetic Beads) | Cell Signaling Technology | | CST#9003S |

**Table S5 The primers and related sequences in the study**

| 1. **Primers and related sequence** | 1. **SEQUCENCE** |
| --- | --- |
| 1. *Lgr5* | 1. Forward: ACCTGTGGCTAGATGACAATGC |
|  | 1. Reverse: TCCAAAGGCGTAGTCTGCTAT |
| 1. *Ascl2* | 1. Forward: AAGCACACCTTGACTGGTACG |
|  | 1. Reverse: AAGTGGACGTTTGCACCTTCA |
| 1. *Adh1* | 1. Forward: GCAAAGCTGCGGTGCTATG |
|  | 1. Reverse: TCACACAAGTCACCCCTTCTC |
| 1. *Aldh1a1* | 1. Forward: ATACTTGTCGGATTTAGGAGGCT |
|  | 1. Reverse: GGGCCTATCTTCCAAATGAACA |
| 1. *Aldh1a2* | 1. Forward: CAGAGAGTGGGAGAGTGTTCC |
|  | 1. Reverse: CACACAGAACCAAGAGAGAAGG |
| 1. *Aldh1a3* | 1. Forward: AGCAACCTGAAGAGGGTCAC |
|  | 1. Reverse: CATCGAAGGGGTCTCCAACT |
| 1. *Rdh7* | 1. Forward: TGGGTCGAGTGTCTTTGTGTG |
|  | 1. Reverse: AACCCGCCAGGCTCTATGATA |
| 1. *Cyp26a1* | 1. Forward: CCCGTGATCGCTGAGGAAG |
|  | 1. Reverse: GGGCACGTCAATGGGAAGAG |
| 1. *Il1b* | 1. Forward: TTCAGGCAGGCAGTATCACTC |
|  | 1. Reverse: GAAGGTCCACGGGAAAGACAC |
| 1. *Il6* | 1. Forward: TTTCCTCTGGTCTTCTGGAGTA |
|  | 1. Reverse: CTCTGAAGGACTCTGGCTTTG |
| 1. *Il10* | 1. Forward: CCCTTTGCTATGGTGTCCTTTC |
|  | 1. Reverse: AGGATCTCCCTGGTTTCTCTTC |
| 1. *Tgfb1* | 1. Forward: GAGCCCGAAGCGGACTACTA |
|  | 1. Reverse: GAGCCCGAAGCGGACTACTA |
| 1. *Il12a* | 1. Forward: CTGTGCCTTGGTAGCATCTATG |
|  | 1. Reverse: GCAGAGTCTCGCCATTATGATTC |
| 1. *Il23a* | 1. Forward: AATAATGTGCCCCGTATCCAGT |
|  | 1. Reverse: GCTCCCCTTTGAAGATGTCAG |
| 1. *Aldh1a2 promoter -1850/-1650* | 1. Forward: CCTGCTTGGAAGATGGGGAA |
|  | 1. Reverse: AGGAAGAGCAAACACCCCAG |
| 1. *Aldh1a2 promoter -1400/-1200* | 1. Forward: CCTCTTGCCTCCATCCAGTG |
|  | 1. Reverse: GGTGGGGAGGTAGCTCTGTA |
| 1. *Aldh1a2 promoter -750/-550* | 1. Forward: ACCCGGAGATTCTGACAGGA |
|  | 1. Reverse: GTCTCCTTCCAGCCCTTTCC |
| 1. *A. muciniphila* | 1. Forward: CAGCACGTGAAGGTGGGGAC |
|  | 1. Reverse: CTTGCGGTTGGCTTCAGAT |
| 1. *Universal bacterial primer* | 1. Forward: GTGCCAGCMGCCGCGGTAA |
|  | 1. Reverse: GGACTACHVGGGTWTCTAAT |
